# Hypothalamic Estrogen Signaling and Adipose Tissue Metabolism in Energy Homeostasis

**DOI:** 10.3389/fendo.2022.898139

**Published:** 2022-06-09

**Authors:** Valeria C. Torres Irizarry, Yuwei Jiang, Yanlin He, Pingwen Xu

**Affiliations:** ^1^ Division of Endocrinology, Diabetes, and Metabolism, Department of Medicine, The University of Illinois at Chicago, Chicago, IL, United States; ^2^ Department of Physiology and Biophysics, The University of Illinois at Chicago, Chicago, IL, United States; ^3^ Pennington Biomedical Research Center, Louisiana State University System, Baton Rouge, LA, United States

**Keywords:** estrogen, ERα, ERβ, VMH, energy homeostasis, WAT, BAT

## Abstract

Obesity has become a global epidemic, and it is a major risk factor for other metabolic disorders such as type 2 diabetes and cardiometabolic disease. Accumulating evidence indicates that there is sex-specific metabolic protection and disease susceptibility. For instance, in both clinical and experimental studies, males are more likely to develop obesity, insulin resistance, and diabetes. In line with this, males tend to have more visceral white adipose tissue (WAT) and less brown adipose tissue (BAT) thermogenic activity, both leading to an increased incidence of metabolic disorders. This female-specific fat distribution is partially mediated by sex hormone estrogens. Specifically, hypothalamic estrogen signaling plays a vital role in regulating WAT distribution, WAT beiging, and BAT thermogenesis. These regulatory effects on adipose tissue metabolism are primarily mediated by the activation of estrogen receptor alpha (ERα) in neurons, which interacts with hormones and adipokines such as leptin, ghrelin, and insulin. This review discusses the contribution of adipose tissue dysfunction to obesity and the role of hypothalamic estrogen signaling in preventing metabolic diseases with a particular focus on the VMH, the central regulator of energy expenditure and glucose homeostasis.

## Introduction

Obesity is a health condition characterized by excessive accumulation of body fat and has become a global epidemic (1). Obesity is associated with numerous metabolic complications such as insulin resistance, glucose intolerance, and hyperlipidemia (2–4). Up to date, therapeutic strategies often include changes in diet and exercise. However, these interventions exhibit little long-term impact. Therefore, there is a critical need to understand the influencing factors that regulate fat expansion and identify alternative long-lasting solutions.

Notably, sex plays a key role in adipose tissue distribution and energy balance regulation. Abundant clinical and epidemiological studies show that men and women differ in body fat accumulation and distribution (5–8). Women have more fat accumulated in subcutaneous white adipose tissue (WAT) (8) and have greater metabolic activity in brown adipose tissue (BAT) when compared to men (9). In contrast, males tend to accumulate visceral fat, which promotes metabolic disorders. This sex-specific fat storage is presumed to implicate evolutionary pressures. Women are adapted to store fat with a low lipolytic rate to respond to chronic energetic challenges such as gestation and lactation (10), protecting them against metabolic diseases (11). In contrast, men have evolved to store metabolically active visceral fat depots as energy fuel under short-term energetic challenges such as hunting (10). However, the sex-specific mechanisms that regulate adipose tissue metabolism are not fully understood.

One of the sex steroids accounting for these sex differences is estrogen. Accumulating evidence suggests that estrogen plays a vital role in sex dimorphic control of metabolic diseases and adipose tissue metabolism (12–14). More specifically, brain estrogen signaling has been shown to enhance sympathetic nervous system (SNS) output, promote thermogenesis in BAT, prevent hyperplasia and hypertrophy in WAT, and regulate WAT distribution (15–20). However, the underlying mechanisms by which central estrogen modulates adipose tissue metabolism are not well characterized. Previous studies were mainly focused on the local effects of estrogen in the brain or adipose tissue (21, 22), but the roles of estrogen-initiated crosstalk between brain and fat in determining sex- and depot-specific adipose tissue function have not been well investigated. This review will summarize the existing evidence regarding brain estrogen-initiated inputs regulating adipose tissue metabolism.

## Obesity and Metabolic Syndrome

Individuals are considered obese when their Body mass index (BMI) is higher than 30kg/m^2^ (23). Obesity occurs as the result of energy imbalance when the caloric intake surpasses energy expenditure, which is influenced by diverse behavioral, socioeconomic, and genetic factors (24). Examples of these factors are sedentary lifestyles or limited physical activity, and excessive caloric intake. Improvement of lifestyle by engaging in activities that require physical exercise and reducing the consumption of high fat/carbohydrate foods can positively impact obesity and metabolic syndrome (25–27). Genetic factors also play a crucial role in energy imbalance and excessive adiposity. Genome-wide associated studies (GWAS) had successfully identified 445 single nucleotide polymorphisms (SNPs) and 389 genes associated with obesity (28). Moreover, several SNPs were found to target miRNAs linked with adipogenesis and lipid metabolism (28). Clinical studies in patients with obesity have revealed that mutations in leptin, pro-opiomelanocortin (POMC), and melanocortin 4 receptor (MC4R) are positively associated with hyperphagia, hyperinsulinemia, and excessive adiposity (29–34). Accordingly, several research efforts have focused on developing therapies against obesity by targeting these genes, mainly to enhance satiety and reduce energy intake.

## Adipose Tissue Function

To better understand the pathogenesis of obesity, it is crucial to define adipose tissue function. Adipose tissue is no longer considered only a storage depot for excess energy. This tissue also acts as a regulator of numerous metabolic and endocrine responses. For instance, adipose tissue regulates appetite, thermogenesis, lipid metabolism, sexual reproduction, immunological responses, insulin signaling, and glucose homeostasis. Moreover, adipose tissue is a highly dynamic organ that consists of numerous cell types, including adipocytes, fibroblasts, endothelial cells, immune cells, and adipocyte progenitor cells. These progenitor cells can undergo adipogenesis to create new adipocytes (35). There are two major types of adipose tissue, white adipose tissue (WAT) and brown adipose tissue (BAT). As detailed below, the excessive adiposity observed in obesity results from the dysregulation of both WAT and BAT metabolic function.

### White Adipose Tissue

**Figure 1:**
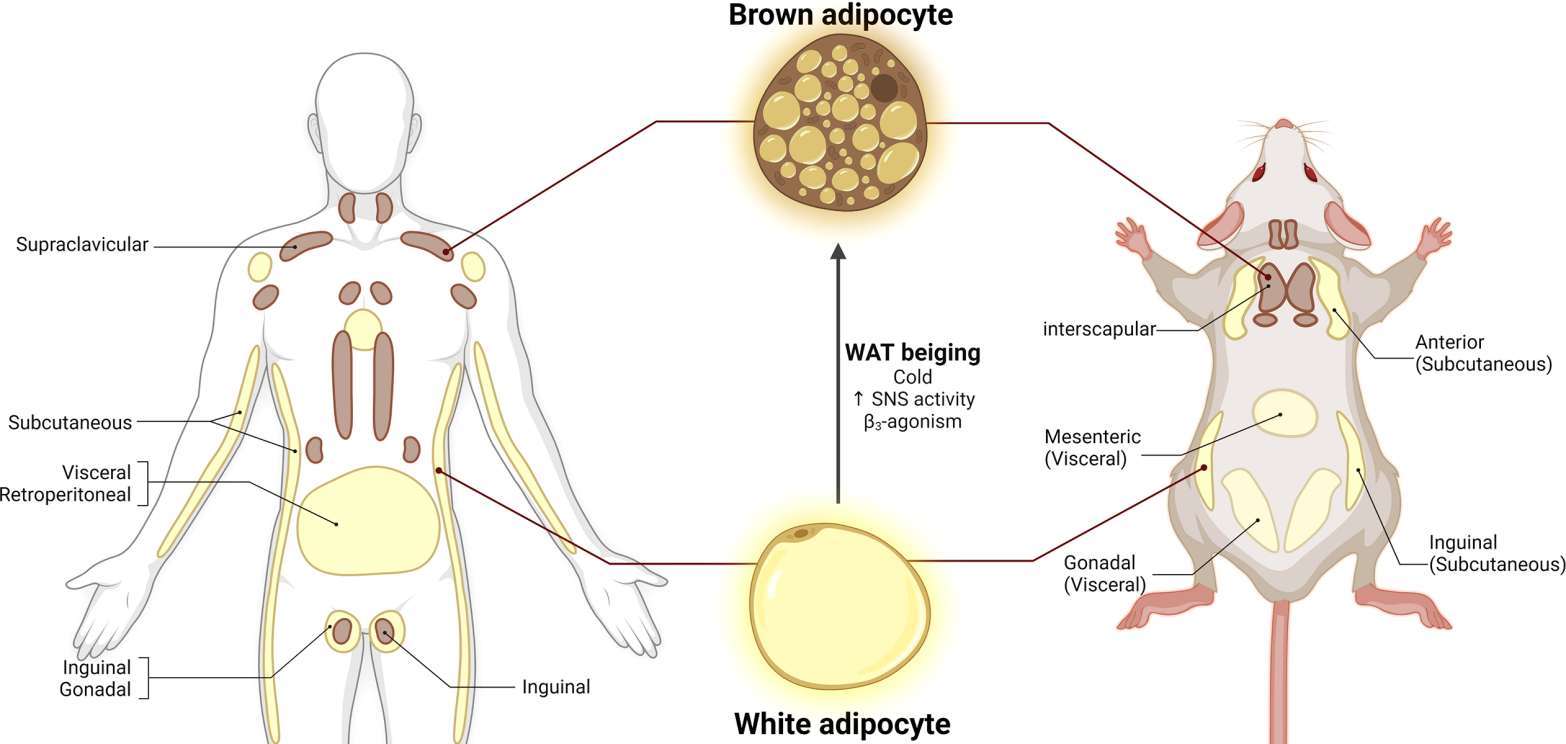
Adipose tissue distribution in humans and mice. White adipose tissue (WAT) is composed of unilocular white adipocytes characterized by a single large lipid droplet. Brown adipose tissue (BAT) consists of brown adipocytes with small lipid droplets and high mitochondrial density. White adipocytes can adopt brown-like morphology under cold exposure, increased SNS activity and β3-agonism, a process called WAT beiging. Adapted from (36). Figure 1 license number: XB23OHGTZA, Figure 2 license number: OZ23OHGU1X.

In living organisms, energy consumption and storage are essential to ensure survival in times of energetic challenges and limited caloric availability. WAT functions as an energy reservoir to store lipids in the form of triglycerides. WAT is mainly composed of white adipocytes, unilocular cells with one large lipid droplet where free fatty acids (FFA) are stored in the form of triglycerides (**Figure 1**). When there is an energy demand for example, after physical activity or under food scarcity, white adipocytes release energy by undergoing lipolysis, a process orchestrated by hormone-sensitive lipase (HSL) and adipose triglyceride lipase (ATGL), where triglycerides are catabolized into FFAs that enter circulation and are delivered to energy-demanding organs such as muscle and liver (35, 37). In addition to its energy storage function, WAT is also an endocrine organ that secrets adipokines such as leptin, adiponectin, and resistin, all of which contribute to food intake and energy balance regulation (38–41)

WAT depots can be further categorized based on their anatomical distribution. Subcutaneous WAT (sWAT) is mainly located in gluteal and femoral regions, whereas visceral/omental adipose tissue (vWAT) is primarily accumulated in the abdomen and internal organs (42, 43). sWAT is associated with optimal metabolic health, whereas vWAT contributes to metabolic dysregulation. For instance, in humans, sWAT expansion is linked to improvement in insulin sensitivity, diminished lipolysis rate, decreased circulation of cytokines, and augmented levels of adipokines (44). In contrast, vWAT expansion is associated with increased insulin resistance, systemic inflammation, and other metabolic syndrome features (45–47). However, it is not clear why vWAT is more metabolically detrimental. It is likely attributed to the anatomical location of vWAT, which is surrounding internal organs within the body cavity. Moreover, vWAT appears to be a highly metabolically active depot, which constantly mobilizes lipids and increases circulating FFA levels. In addition to gender difference, adipose tissue expansion is dependent on genetic components and diet (48). White adipocytes can increase in number (hyperplasia) or size (hypertrophy) in response to excessive energy intake (48, 49). While WAT is highly plastic that can expand from 4% to 50-70% of an individual’s body composition, the total capacity is limited. When WAT expansion goes beyond its capacity, excess fat begins to accumulate in tissues, such as skeletal muscle, liver, heart, and pancreas, ultimately leading to lipotoxicity, systemic inflammation, insulin resistance, and metabolic disorders (50–52)

### Brown and Beige Adipose Tissue

In contrast to WAT, BAT is a highly vascularized organ mainly composed of brown adipocytes. Brown adipocytes have small lipid droplets and are rich in mitochondrial content, which contributes to their brown appearance (**Figure 1**) (53, 54). The primary function of BAT is thermogenesis, a process by which the uncoupling protein 1 (UCP1, expressed in the inner mitochondrial membrane) uncouples oxidative phosphorylation to dissipate energy as heat (54–56). In both humans and rodents, BAT thermogenesis is mainly dependent on the sympathetic nervous system (SNS) activation (55–59). Activation of SNS drives the release of norepinephrine (NE) which activates β3-adrenoceptors (β3-AR) expressed in brown adipocytes. Mitochondria typically generate energy by synthesizing ATP *via* oxidative phosphorylation. However, β3-AR activates the cyclic AMP/Protein kinase A pathway to release FFA, which will be used as fuel by UCP1 to uncouple ATP synthesis and release the produced energy in the form of heat (55, 58). BAT thermogenesis can be induced by multiple factors such as diet, cold exposure, and β-adrenoceptors agonists (53, 57, 58, 60–63). In addition to UCP1, other important thermogenic and BAT markers have been identified including peroxisome proliferator-activated receptor gamma coactivator 1-alpha (PGC1α), PR domain containing 16 (PRDM16), cell death-inducing DFFA-like effector A (CIDEA), and type II iodothyronine deiodinase (DIO2) (20, 57, 59, 64). It was once thought that BAT was absent in adulthood. However, positron emission tomography-computed tomography (PET/CT) scanning technologies with ^18^F-fluorodeoxyglucose (^18^FDG) have revealed that adult humans have metabolically active BAT depots (9, 56, 61, 62). BAT content in humans can be found in cervical supra-clavicular, axillary, and paraspinal regions, accounting for approximately 2% of total fat depots (**Figure 1**) (9, 61). As an endocrine organ, BAT secretes batokines as well as cytokines such as fibroblast growth factor 21 (FGF21) and interleukin 6 (IL-6) (65, 66). However, the endocrine functions of BAT in physiological and pathological conditions still need to be fully explored.

In addition to its thermogenic and endocrine function, BAT also plays an essential role in glucose homeostasis. BAT glucose uptake can be stimulated by SNS-activated thermogenesis or insulin signaling (67). BAT expresses insulin receptors that allow translocation of GLUT4 into the plasma membrane, thus facilitating glucose clearance (35, 67). Studies have shown that human-to-mice BAT transplantation results in increased insulin sensitivity, decreased body weight gain, and attenuated high fat diet (HFD)-induced insulin resistance (66). In line with this, fasting-induced insulin resistance is accompanied by decreased BAT glucose uptake and thermogenic activity in humans (68). These findings provide additional potential connection between BAT dysfunction and metabolic syndrome.

Under specific physiological stressors, brown-like adipocytes can arise from white adipocytes or adipocyte progenitor cells. These cells are termed beige/brite adipocytes and the process by which they emerge is called WAT browning or beiging. Beige adipocytes share morphological features with both white and brown adipocytes. Beige adipocytes have larger lipid droplets for fat storage, and although they have less mitochondrial density, they are UCP1 positive cells and they possess thermogenic capacity (69, 70). Similar to BAT activation, WAT beiging can be induced by cold exposure, physical activity, and β-adrenergic receptors (**Figure 1**) (57, 63, 71). BAT thermogenesis and WAT browning are potential therapeutic targets to treat obesity and metabolic syndrome because they can consume glucose and fatty acid. Cold exposure in humans results in increases in BAT content, thermogenic activity, and energy expenditure, accompanied by lower BMI and body fat content (61, 72, 73). Conversely, BAT content was lower in individuals with higher visceral fat deposition and BMI (72). Additionally, individuals with detectable active BAT depots display lower circulating cholesterol and glucose levels (74). In rodents, UCP1 ablation leads to body weight gain, increased food intake, and decreased diet-induced thermogenesis, phenotypes that are exacerbated by HFD (75). Activation of β3-AR using CL316,243 agonist remodeled white adipocytes into more multilocular structures and smaller lipid droplets, associated with increased expression of brown adipocyte markers and UCP1 protein (57). Murano et al. reported an increase in noradrenergic fiber density in BAT and WAT following 10 day-cold exposure in adult female mice (63). The abundant evidence from the animal and clinical studies strongly support that increasing BAT activity and inducing WAT beiging represent viable strategies to prevent and treat obesity and its related comorbidities.

However, clinical evidence on BAT activation contribution to body weight loss is scarce. To this date, there are more than 40 ongoing clinical trials aiming to better understand chronic BAT activity in humans and test potential therapeutic approaches. In healthy males, long-term cold exposure (17°C, 2 hours/day for 6 weeks) stimulated BAT activity, increased energy expenditure, and significantly reduced body fat mass (76). Recently, mirabegron, a pharmacological agent and β3-adrenergic receptor agonist, has been tested in both men (NCT01783470) and women (NCT03049462). Healthy men receiving 200 mg mirabegron (3 interventions within 28 days) showed increased acute BAT activation and resting metabolic rate (RMR) (77). On the other hand, healthy women received daily 200 mg mirabegron for 28 days, resulting in enhanced BAT metabolic activity, increased resting energy expenditure, and improved insulin sensitivity. However, no changes in body weight mass were detected in these women (78). These findings position mirabegron as a promising therapeutic agent to stimulate endogenous BAT thermogenic activity in patients with metabolic syndrome. Further research is needed to identify the cellular, molecular, and transcriptional mechanisms governing the BAT/beige development and function.

## Sexual Dimorphism in Adipose Tissue

Sex-specific distribution of adipose tissue has been described in humans and animal models. Females are more abundant in sWAT, while males tend to have more abdominal-visceral depots (79, 80). Males have evolved to store highly metabolically active visceral fat depots that can be quickly mobilized and used as energy fuel under short-term energetic challenges such as hunting. On the other hand, females might have evolved to store fat subcutaneously with low lipolytic rates, which would store the energy for long-term challenges such as pregnancy and lactation (10). This evolutionary adaptation might confer protection against obesity and metabolic syndrome. Indeed, in studies using mice exposed to HFD for twelve weeks, males tend to gain more body weight and have higher expression of pro-inflammatory genes in the adipose tissue when compared to females (81). In contrast, women with vWAT accumulation are at higher risk of developing impaired glucose and lipid metabolism and cardiovascular complications when compared to men (7, 8). Clinical studies have shown that catecholamine-induced lipolysis is predominant in female but not male abdominal adipocytes (82). Additionally, during fasting, while FFA increases in the blood from both sexes, women have lower levels of circulating glucose when compared to men. This diminished glucose production is speculated to protect women from FFA-induced insulin resistance (83). Interestingly, a study addressing sex-specific responses to exercise revealed that women derived more energy expenditure from fat oxidation. In contrast, men utilized carbohydrates as the primary fuel during the exercise sessions (84). Taken together, these studies suggest that a higher lipolytic rate in women is possible because they are more dependent on fat as an energy source.

Importantly, the protective role of sWAT in females seems to be age-dependent, as postmenopausal women suffer fat redistribution. Fat depots from subcutaneous regions are transferred to visceral regions (85). This has led to the conclusion that sex hormones might play a critical role in gender-specific fat distribution and overall metabolic health. Particular attention has been given to estrogen as it has been shown that the decreased circulation of estrogen contributes to increased adiposity, insulin resistance, low metabolic rate, and adipose tissue inflammation (59, 86, 87).

One proposed mechanism for gender-specific fat distribution is the estrogens-mediated modulation of sympathetic inputs and lipolytic/lipogenic rates in adipose tissues. In human WAT, adrenergic activation of β-adrenergic receptor (AR) promotes lipolysis, whereas adrenergic stimulation of α2A-AR inhibits lipolysis (88, 89). As discussed earlier, sWAT in women is characterized by a lower lipolytic rate to ensure energy preservation. In line with this, healthy women under long-term estradiol (E2) treatment have increased α2A-AR mRNA levels and protein binding capacity in subcutaneous depots compared to placebo-treated individuals. E2 did not affect the α2A-AR mRNA profile in vWAT (88). *In vitro* studies with cultured adipose tissue fragments consistently show that E2 treatment exerts anti-lipolytic effects in subcutaneous adipocytes (88). This suggests that estrogens modulate female WAT distribution in part by upregulating α2A-AR signaling in sWAT while stimulating β-AR-mediated high lipolytic rate in vWAT, thus promoting subcutaneous energy storage.

Moreover, these previous findings were associated with estrogen receptor alpha (ERα), which has been evidenced to be the main target for estrogen’s anti-obesity effects (further discussed below) (88). In line with this, mutations in ERα are linked with higher BMI, waist circumference, and increased fat mass in middle-aged women (90). In rodents, central E2/ERα signaling has been shown to contribute to changes in fat distribution. Deletion of ERα in the VMH SF1 neurons leads to increased body weight, massive gonadal WAT (gWAT, rodent visceral adipose depot) expansion, increased lipogenesis, and decreased sympathetic tone (20). The role of estrogen in the regulation of energy balance will be discussed in more detail in the following section.

Similar to WAT, sex differences in BAT have also been described. The volume of BAT depots between the sexes is similar. However, women have higher BAT mass in the cervical-supraclavicular region when compared to men, and female BAT is metabolically more active than males (9, 61, 91). Cold exposure results in significantly higher energy expenditure in women (61). Interestingly, the higher prevalence of metabolically active BAT in women is positively correlated with plasma estradiol levels (92, 93). Treatment of β3-adrenergic receptor agonist CL316,243 (CL) upregulates thermogenic markers and mitochondrial respiratory chain proteins in gWAT of female mice but not in males, indicating sex-specific WAT browning phenotypes (57). Female rats have higher BAT activity and oxygen consumption than males under *ad libitum* conditions. However, under caloric restriction, these phenotypes are reversed, as indicated by diminished mitochondrial density and downregulation of thermogenic genes in females (94).

A recent report by MacCannell et al. used high-resolution respirometry to assess sex-specific mitochondrial function and metabolic flexibility in mice challenged with HFD. HFD-fed females show increases in electron transport chain (ETC) components, specifically Complex I and II respiration, in BAT mitochondria compared to females fed a standard diet, suggesting adaptive mitochondrial BAT respiration. Notably, HFD-fed males did not exhibit this phenotype. In addition, improved mitochondrial respiration is accompanied by upregulation of BAT-associated genes. These findings show that under HFD condition, female mice have greater metabolic flexibility and adapt to nutritional challenges by maximizing the activity of ETC complexes to modulate energy expenditure and restore energy balance (95). Altogether, these findings provide evidence for sex-specific regulation of BAT metabolism and suggest mitochondrial metabolic flexibility as a potential mechanism underlying the physiological relevance of BAT function in women.

## Estrogen

Estrogen is a sex hormone mainly produced in the ovaries, corpus luteum, placenta, and other non-gonad regions like the heart, liver, and brain (96). In females, the three types of estrogen present are estrone (E1), estradiol (E2/17β-estradiol), and estriol (E3). Estrogen biosynthesis initiates with cholesterol. A series of chemical reactions result in the synthesis of testosterone. The action of aromatase mediates the conversion from testosterone to estradiol (96). E2 or 17β-estradiol is the most active form of estrogen in premenopausal women. The levels of estrogen are decreased post-menopause, which is associated with a higher risk of metabolic complications and body weight gain. The anti-obesity effects of estrogen and its role in energy homeostasis are well evidenced. For instance, Hong et al. demonstrated that compared to females, male mice were more susceptible to increased body fat. However, women begin to experience body weight gain after menopause (97). Importantly, ovarian hormone depletion by OVX removed female mice protection against body weight gain (86). In line with this, hormone replacement therapy in postmenopausal women or E2 replenishment in OVX mice reverts the obesogenic phenotypes (97, 98). It has also been reported that food intake and body weight gain in rats varies depending on the estrous cycle stage, pregnancy, or lactation (59). In female mice, disrupted estrogen signaling leads to obesity, decreased locomotion, and reduced heat production and energy expenditure (19), demonstrating estrogen’s impact on thermogenesis. Estrogen signaling is mediated by its receptors, Estrogen receptor alpha (ERα), Estrogen receptor beta (ERβ), G protein-coupled receptor 30 (GPR30) and Gq protein-coupled receptor (Gq-mER), all of which are expressed in the brain and adipose tissue (96).

### ERα

From all the estrogen receptors, ERα is the most extensively studied in the context of energy homeostasis, given the abundant evidence suggesting that ERα mediates estrogen’s anti-obesity effects. ERα is expressed in gonadal organs, liver, adipose tissue, and brain. In the brain, ERα expression predominates in the bed nucleus of the stria terminalis, the nucleus tractus solitarius (NTS), amygdala, hypothalamus, periaqueductal gray and dorsal raphe nucleus (DRN) (99). Specific hypothalamic regions that express ERα include POMC neurons and neuropeptide Y (NPY)/agouti-related protein (AgRP) neurons, which are found in the arcuate hypothalamic nucleus (ARH), ventromedial hypothalamic nucleus (VMH) and medial preoptic area (MPOA) (99). All these brain regions are implicated in food intake and thermoregulation. Mutations in *ESR1*, the gene that encodes ERα, have been associated with higher BMI and increased fat mass in women (90). In rodents, ERα congenital global KO results in increased adiposity and elevated serum glucose and insulin in both male and female mice (100). Consistently, a point mutation of the palmitoylation site of ERα (C451A-ERα), which results in membrane-specific loss of function, impaired glucose sensing in female mice (101), and disturbed sexual differentiation in the perinatal programming of the male brain (102). Depletion of ERα in the mouse brain abolishes the beneficial metabolic effects of estrogen, resulting in hyperphagia, body weight gain, increased visceral adiposity, and impaired energy expenditure (17, 20, 59, 103). In human adipose tissue, expression of ERα is positively associated with UCP1 protein density (80). Similarly, adipose tissue ERα expression in mouse is correlated with lower adiposity, higher UCP1 content, optimal BAT thermogenic function, and insulin sensitivity (104). Activation of ERα with synthetic agonist propylpyrazoletriol (PPT) upregulates UCP1 and causes WAT beiging both *in vitro* and *in vivo* (103).

### ERβ

The role of ERβ in adipose tissue function is more controversial. This receptor is primarily expressed in bone marrow, endothelium, lungs, adipose tissue, and the brain. In the brain, ERβ is most abundant in the hippocampus, bed nucleus of the stria terminalis, amygdala, DRN, and very limited in the hypothalamus (99). ERβ has not been paid as much interest as ERα, not only because of the evidence discussed earlier favoring ERα as the principal mediator of estrogen signaling, but also because most studies targeting ERβ have concluded that it does not affect energy homeostasis. For example, depletion of ERβ in animals has no effect on total adiposity in mice (105). In OVX rats, activation of ERα by its agonist propylpyrazole triol (PPT) causes body weight loss and hypophagia associated with a reduction in meal size. However, using diarylpropionitrile (DPN) to activate ERβ does not influence body weight and food intake (98, 106). In contrast, recent reports have shown that ERβ might have a beneficial impact on metabolic health and potentially a protective function. González-Granillo et al. recently demonstrated that subcutaneous injection of ERβ agonist 4-(2-(3,5-dimethylisoxazol-4-yl)-1H-indol-3-yl)phenol (DIP) induces fat redistribution from visceral to subcutaneous depots, elevates heat production, and stimulates WAT browning in male mice (107). Similarly, activation of ERβ using a synthetic agonist (b-LGND2) decreases body weight and fat mass in WT mice. Deletion of ERβ reversed these phenotypes (108).

It has also been reported that ERβ is more abundant in healthy females when compared to men. Recently Porter and colleagues studied the transcriptional and protein content of estrogen receptors in obese individuals, including men, premenopausal and postmenopausal women. They report that ERα and ERβ protein expression in adipose tissue was positively correlated with UCP1 content, further evidencing the role of ERα in BAT thermogenesis and suggesting that ERβ may also play an important part. Importantly, they showed that ERβ gene expression was higher in adipose tissue of women when compared to men (80). It has been suggested that in the absence of ERα, the available estrogen (produced in adipose tissue, the second primary source of estrogen after ovaries) is forced to activate ERβ as compensatory mechanism, indicating that ERβ may have a protective metabolic role in the absence of estrogen or ERα (109). However, further studies are required to clarify the discrepancies in the field and elucidate the potential role of ERβ in metabolic health.

### GPR30

Although ERα and ERβ are the most studied ERs in the context of energy homeostasis, there are other novel receptors that represent a promising therapeutic target for metabolic syndrome. The GPR30 or G protein-coupled estrogen receptor 1 (GPER1) is expressed in reproductive organs, adipose tissue, pancreas, bone tissue, and brain, including the hypothalamus (110–114). The binding of estrogen to GPR30 initiates a signaling cascade that activates the protein kinase A (PKA)/ERK pathway (113).

Although GPR30 has a lower binding affinity to estradiol when compared to ERα (113), various reports employing a whole-body deletion model (GPR30 KO) suggest that this receptor is essential for the maintenance of glucose and energy balance. Deletion of GPR30 in mice leads to increased body weight attributed to increased fat mass, augmented circulating levels of cholesterol and triglycerides, glucose intolerance, impaired insulin sensitivity, and elevation of proinflammatory cytokines (115). Consistent with these findings, Davis et al. reported that whole-body GPR30 KO mice show elevated body weight, increased adipocyte size, BAT remodeling, and decreased UCP1 gene expression and energy expenditure (112).

Interestingly, male mice exhibit more susceptibility to GPR30 deletion when compared to females, as male GPR30 KO mice show significant changes in body weight as early as 8 weeks of age, but GPR30 KO females begin to significantly gain weight at 13 weeks of age. In addition, adipocyte area and UCP1 levels are not significantly changed in female GPR30 KO mice as opposed to male mice. In the same study, GPR30 KO females were ovariectomized and treated with E2. Estradiol supplementation did not affect body weight changes, glucose tolerance, and adipocyte morphology in these GPR30 KO OVX mice. These findings imply that GPR30 in males is required to maintain optimal body weight and energy expenditure, and in females, it confers protection against adipose tissue remodeling and glucose imbalance (112).

It is important to note that in female mice, deletion of GPR30 decreased hypothalamic ERα and increased ERβ gene expression, suggesting that GPR30 may regulate the expression of classical ERs in the hypothalamus (112). Female mice lacking GPR30 also show hyperglycemia and decreased insulin expression and release from the pancreas (114). In contrast to these findings, another report indicates that GPR30 KO female mice fed an HFD exhibit decreased body fat mass, improved glucose balance and insulin sensitivity, reduced adipocyte size and adipogenesis, indicating that in HFD-fed females, GPR30 contributes to adipose tissue expansion and energy imbalance (110). Recently, Sharma et al. reported that GPR30 agonism with GPR30-selective small-molecule agonist G-1 reverses OVX-induced obesity by decreasing body weight and fat mass, increasing energy expenditure, promoting BAT remodeling, improving glucose homeostasis, and lowering pro-inflammatory responses. These phenotypes persisted in OVX mice challenged with HFD, demonstrating that GPR30 activation exerts anti-obesity effects in female mice (116).

### Gq-mER

The Gq protein-coupled receptor (Gq-mER) has gained attention over the past years, but limited evidence links this membrane ER with energy balance. Gq-mER is mainly expressed in the hypothalamus, including both NPY/AgRP and POMC neurons in ARH, the paraventricular nucleus (PVN), and the MPOA (117–122). Early studies demonstrate the expression and activation of hypothalamic Gq-mER, as administration of STX (Gq-mER selective ligand) induces POMC neural responses in ERαKO, ERβKO, and GPR30KO mice (117, 122). In addition, STX mimics the effect of E2 replacement in OVX guinea pigs by decreasing body weight and uterine weight (122). The suggested mechanism by which Gq-mER regulates neural responses in ARH is through GABA_B_ receptors expressed in NPY/AgRP and POMC neurons. Gq-mER activation desensitizes GABA_B_ receptors, increasing membrane excitability in POMC neurons, while in NPY/AgRP neurons, it enhances GABA_B_ inhibitory effects, indicating that Gq-mER agonism triggers anorexigenic signals (118, 123). In line with this, daily subcutaneous injection of STX results in decreased food intake in gonadectomized male and female guinea pigs (118). Gq-mER signaling has also been associated with the regulation of body temperature. Treating OVX guinea pigs with STX results in reduced core body temperature accompanied by decreased fat pad weight and body weight and reductions in food intake and meal frequency (121). In summary, Gq-mER has been shown to play an essential role in estrogenic regulation of energy balance. Further research is required to identify the potential therapeutic applications of these ERs.

## Estrogen in the Brain

In 2011, we provided genetic evidence that brain ERα signaling is vital for body weight control. In this study, we crossed ERα^flox/flox^ with Nestin-Cre mice to selectively delete ERα from CNS (ERα^CNS^-KO). We found that compared to their littermate controls, both male and female ERα^CNS^-KO mice have increased body weight, which is solely reflected by an increase in fat mass, but not lean mass. In addition, we found these mice eat more and have decreased energy expenditure accompanied by reduced physical activity. Importantly, we showed that ERα^CNS^-KO mice have a larger visceral fat depot than the controls, which in other words, they have abdominal obesity. Finally, we found that ERα^CNS^-KO females have increased E2 in the blood (20). This is important when put in a broader perspective, as it indicates that increased peripheral estrogen signaling does not rescue obesity induced by brain-specific KO of ERα, suggesting an essential role of brain ERα in body fat distribution and energy expenditure.

### ARH

Fluctuations in feeding behavior are estrous cycle-dependent in female rodents. Specifically, food intake elevates during the metestrous-diestrous phase, when estrogen levels are at the lowest. Conversely, food intake decreases to the lowest point during the proestrus-estrous stage, in which estrogen levels are high (124–126). Notably, the reduced food intake and body weight during the proestrus phase is associated with decreased NPY/AgRP and increased POMC mRNA expression, suggesting a potential role of ARH appetite-regulating neuropeptides in estrogenic regulation of food intake (125). Consistently, POMC excitatory synapses are enhanced during the proestrus phase (126). E2 supplementation in OVX mice decreases appetite and adiposity, associated with enhanced POMC neuronal activity, as indicated by increases in POMC excitatory synapses and c-fos expression (126). ERα congenital globe KO lowers POMC immunoreactivity, which was not reversed by E2 replenishment, suggesting that POMC synaptic plasticity is mediated by E2/ERα signaling. These findings support a model that estrogens modulate food intake by inhibiting AgRP neurons and activating POMC neurons, resulting in an anorexigenic response (126).

In supporting this point, administration of E2 directly into the ARH fails to decrease food intake in mice lacking AgRP/NPY neurons (121), suggesting a vital role of AgRP/NPY neurons. However, although ERα is the primary mediating receptor for anorexigenic effects of estrogens, ERα does not express in AgRP/NPY neurons (121). These findings indicate that estrogens may act through ERα expressed by presynaptic neurons to regulate AgRP/NPY neurons. Consistently, the following study demonstrated that the anorexigenic effects of E2 are blocked in female ERα^flox/flox^/POMC-Cre mice with ERα selectively deleted from POMC progenitor neurons (120), implying a mediating role of ERα expressed by POMC progenitor neurons. Mechanistically, phosphatidylinositol 3-kinase (PI3K) has been suggested to mediate the metabolic functions of ERα signals. Specifically, estrous cycle-dependent fluctuations in food intake are blunted in female mice with PI3K genetically inhibited in the POMC progenitor neurons (124). These results indicate that an ERα-PI3K cascade in POMC progenitor neurons mediates estrogenic actions to suppress food intake.

In line with this, ablation of ERα, specifically in POMC progenitor neurons, leads to chronic hyperphagia, decreased leptin sensitivity, and body weight gain in female mice (20). Interestingly, a recent study showed that region-specific deletion of ERα in the ARH of ERα^flox/flox^ mice by stereotaxic delivery of AAV virus has no effect on food intake, suggesting that estrogen may exert its anorexic effects by targeting other brain sites rather than the ARH (16). This discrepancy could be due to the non-ARH deletion of ERα in the ERα^flox/flox^/POMC-Cre model. The recombination in off-target sites could contribute to physiological phenotypes of POMC-Cre transgenics (127). It is possible that ERα deleted in NTS POMC neurons led to changes in food intake.

### NTS

The NTS is a brainstem region that regulates satiety (128). E2 treatment has been shown to increase neuronal activity (as measured by c-fos expression) in the NTS of OVX rats. Moreover, c-fos expression colocalized with ERα in the NTS of E2-treated rats (129). Expression of ERs in the NTS of OVX rats is increased following 48-hour fasting (130). It has also been demonstrated that ERα mRNA expression in the NTS of OVX rats fluctuates with the estrous cycle, showing higher expression during estrous and lower expression during proestrus. OVX rats exhibit reduced ERα NTS mRNA expression compared to sham-operated rats (131).

### DRN

The DRN is the primary site for 5-hydroxytryptamine (5-HT, serotonin) synthesis in the brain. Interestingly, 90% of the serotonergic neurons in the DRN (5-HT^DRN^) coexpress ERα (132). Ovarian hormone depletion in female rats results in decreased c-fos expression in 5-HT^DRN^ neurons. Conversely, E2 replenishment increases serotonergic neural activity in the DRN (133). Specific deletion of ERα in the DRN of OVX mice results in binge-like eating behavior, and estrogen replacement fails to reverse this phenotype. Moreover, activation of 5-HT by either E2 or PPT suppresses binge-like eating (132). A study in OVX rats by Santollo et al. reported that food intake was significantly decreased within 24 hours after specific E2 microinfusion in ARH, DRN, and the MPOA (134).

### MPOA

The MPOA has been identified as a major thermoregulatory hypothalamic region. MPOA subregions show either cold-sensing or warm-sensing properties in sham female rats. However, after estrogen depletion by OVX, c-fos signaling is significantly decreased, and thermosensitivity of MPOA subregions is impaired (135). The MPOA has also been linked with torpor regulation in rodents. A recent study showed that activation of ERα^+^ neurons in MPOA decreases core body temperature and lowers energy expenditure and metabolic rate. Furthermore, ablation of ERα in MPOA reverses the torpor-like state in females but not in males (136). These findings suggest an integrating role of MPOA estrogen signaling in the regulation of energy and temperature homeostasis. Besides estrogens, tamoxifen, a selective estrogen receptor modulator, has also been shown to modulate the metabolic functions of MPOA neurons. For example, subcutaneous injection of tamoxifen alters the transcriptional profile in all MPOA cell types by repressing metabolic genes. Hypothalamic ablation of ERα using ERα^flox/flox^/Nkx2-1-Cre mice, resulting in ERα deletion in MPOA, vlVMH, and ARH, eliminates the tamoxifen-mediated gene repression and upregulates metabolic genes in the MPOA. In line with this, hypothalamic ablation of ERα blunts tamoxifen-induced inhibition of BAT thermogenesis and locomotion. These findings suggest that tamoxifen acts through ERα expressed by the MPOA neurons to impair energy expenditure and suppress metabolism-related genes (137).

### Sagittalis Nucleus of the Hypothalamus (SGN)

SGN, a unique hypothalamic nucleus discovered in 2008, is located between the ARH and VMH. Interestingly, this novel hypothalamic region was rich in ERα-immunoreactive cells (138). Studies focusing on SGN’s role in energy balance are very limited. C-fos immunoreactivity was higher in the SGN of OVX rats treated with estradiol when compared to control OVX rats. In line with this, neural activity is enhanced when rats of both sexes engage in sexual behavior, supporting the notion that ERα^SGN^ might play a role in sex-specific sexual arousal (139, 140). Further research might provide a better understanding of the physiological relevance of ERα^SGN^ in sexual behaviors.

### VMH

ERα signaling in the VMH has also been shown to modulate thermogenesis and glucose homeostasis. Silencing ERα in VMH leads to hyperphagia, hyperglycemia, and decreased energy expenditure (20). The role of estrogen signaling in VMH will be discussed in more depth later in this article.

## Central Estrogen Interaction with Hormones, Adipokines, and Kinases

### Leptin

Leptin is an adipokine synthesized in adipocytes and encoded by the obesity (*ob*) gene with anorexigenic properties. Leptin is considered a biomarker for obesity as the levels of leptin are directly proportional to body fat mass accumulation (141). Leptin signals through leptin receptors (LepR, encoded by *db* gene) in the brain to communicate the status of body fat stores. If fat depots are not metabolized, leptin will inhibit NPY/AgRP neurons and activate POMC neurons to exert an anorexigenic response and increase satiety (141). During LepR activation, JAK2 tyrosine kinase phosphorylates LepR, which initiates the recruitment and activation of the STAT3 signaling cascade. STAT3 is essential for leptin signaling, as deletion of STAT3 leads to hyperphagia and impairs energy expenditure (142). Mutations in the LepR-B gene results in obesity in humans and rodents (128). Leptin-deficient *ob/ob* mice receiving intraperitoneal injections of leptin experience decreases in body mass and food intake compared to *ob/ob* mice receiving PBS treatment (143). Estrogen has been reported to be a key determinant of serum leptin levels and central leptin sensitivity. In diabetic Akita female mice –mice carrying *Ins2* mutation–, ERα ablation exacerbates hyperphagia by further decreasing central leptin signals and downregulating POMC gene expression (144). OVX rats display dramatic increases in serum leptin levels, associated with significant changes in body weight gain. After E2 replacement, serum leptin levels are decreased (145, 146). Both *ob/ob* and *db/db* mice treated with E2 for 4 weeks showed body weight loss, diminished fat mass, hypophagia, and energy expenditure. This was accompanied by elevated hypothalamic pSTAT3 and increased POMC immunoreactivity (126). In the hypothalamus, LepR has been shown to coexpress with ERα and ERβ, particularly in the MPOA, PVN, dorsomedial hypothalamus (DMH), VMH and ARH (142). Interestingly, estrogen depletion by OVX decreases leptin gene expression in these hypothalamic regions (147). Similarly, E2 replenishment in OVX rats significantly increased hypothalamic leptin content, accompanied by decreases in energy intake and body weight (145). Deletion of LepR in vagal afferent neurons was shown to increase body weight gain, fat accumulation, adipocyte size, and meal numbers. These obesogenic phenotypes were associated with lower circulating estrogen and decreased expression of ERα in vagal afferent neurons (148). Altogether this evidence shows that central estrogen signaling interacts significantly with leptin-induced anorexigenic signals.

As a hormone that directly interacts with hypothalamic neurons to modulate WAT distribution and energy intake, it is not surprising that leptin has also been reported to act on thermoregulating brain regions to influence body temperature and BAT thermogenesis. In addition to ARH, leptin-sensitive neurons can be found in the MPOA, PVN, DMH, VMH, and lateral hypothalamus (LH) (149). These brain regions are well known for their thermoregulatory and thermogenic properties. *Ob/ob* mice display decreased body temperature at thermoneutrality, which is brought back to basal levels following leptin administration (150). Leptin deficiency affects body temperature during thermoneutral conditions and when being challenged by cold temperatures. Mice lacking both leptin and UCP1 (*ob/ob*.Ucp1^−/−^) are unable to survive temperatures below 12°C. Interestingly, leptin treatment allowed *ob/ob*.Ucp1^−/−^ mice to adapt to cold exposure by increasing their core temperature and oxygen consumption (151). Leptin has been shown to upregulate Ucp1 mRNA levels as well as thermogenic and sympathetic markers in rodents with intact BAT sympathetic innervation (152, 153).

A clinical study performed in pre- and post-menopausal women evaluated the relationship between ERα/ERβ ratio and obesity. They showed that a lower ERα/ERβ ratio in omental adipose tissue (vWAT) is positively correlated with higher BMI and waist/hip/thigh circumference. Moreover, serum leptin levels significantly correlated with these obesity markers, suggesting that circulating leptin may mediate the regulatory effects of estrogen signaling on adipose tissue homeostasis (154). Similar to leptin, estrogen’s effect on BAT thermogenesis, thermoregulation, cold adaptation, and energy expenditure has been evidenced (9, 20, 57, 59, 61, 87, 136, 155). As discussed later in this article, estrogen and leptin were identified as energy sensors that signal through the AMPK(VMH)-SNS-BAT axis to activate BAT thermogenesis (59, 156). Additional studies might be valuable to elucidate a potential estrogen-leptin interaction and its effects on energy expenditure and overall metabolic health.

### Ghrelin

Ghrelin is an orexigenic hormone produced in the stomach that stimulates food intake *via* its growth hormone secretagogue receptors (GHSRs), which are expressed in the ARH and VMH (157). In contrast to leptin, ghrelin is secreted under fasting conditions and signals through GHSRs to activate NPY/AgRP neurons and inhibit POMC neurons, leading to an orexigenic response and an increase in food intake (155).

In estrogen-depleted rats, ghrelin mRNA in the stomach is increased, suggesting that the absence of ovarian hormones facilitates ghrelin synthesis. In line with this, ERα was co-expressed with ghrelin in an intact female rat stomach. E2 replenishment in OVX rats decreases plasma ghrelin levels and mRNA content in the stomach (158). Ghrelin-induced increases in food intake are tightly associated with the estrous cycle in female rats. Intracerebroventricular (ICV) administration of ghrelin results in increased energy intake principally during the diestrus cycle when estrogen levels are lower. Consistently, after ghrelin ICV infusion, neural activity is greater in ARH during the diestrus phase rather than the proestrus phase (159). Yokota-Nakagi et al. explored the effects of estrogen on ghrelin effect in HFD-fed OVX rats. As expected, estrogen depletion accompanied by HFD dramatically increased body weight, food intake, and vWAT. Implantation of E2 pellets reversed the obesogenic phenotypes. Administration of GHRP-6, a GHSR agonist, increased HFD intake in OVX mice treated with placebo pellets, but those mice with E2 replenishment were protected from the orexigenic effects of GHRP-6. Similarly, E2 decreased c-fos expression in ARH following GHRP-6 administration (155). These findings suggest that estrogen influences ghrelin signaling in ARH neurons.

Although ghrelin is widely known for its effects on energy intake, multiple reports have evidenced the role of ghrelin in energy expenditure. Lin et al. worked with old Ghsr^−/−^ mice to show that ablation of ghrelin receptor decreases body weight and improves insulin sensitivity. These phenotypes were induced by energy expenditure rather than changes in food intake or physical activity. Specifically, the increase of energy expenditure in Ghsr^−/−^ mice is accompanied by increases in O_2_ consumption, CO_2_ production, RMR, and respiratory quotient (RQ). *In vitro* and *in vivo* studies demonstrated that GHSR ablation results in increased UCP1 content and upregulated thermogenic genes in BAT (160). We showed that neuron-specific deletion of GHSR partially prevents DIO in mice by improving insulin sensitivity and metabolic flexibility, increasing energy expenditure and physical activity. DIO-neuron-deficient GHSR mice demonstrated higher body core temperature and resistance to cold exposure when compared to control mice. Interestingly, expression of GHSR was upregulated in the ARH and VMH of wild-type mice fed an HFD when compared to mice fed a regular diet, suggesting that ghrelin signals through these hypothalamic neurons to counteract the effects of HFD (157). The potential role of ovarian hormones (including estrogen) in ghrelin-regulated energy expenditure remains to be elucidated.

### Insulin

Adipose tissue dysfunction and obesity are tightly linked to insulin resistance and diabetes (161). Insulin is a hormone synthetized and secreted by β-cells in the pancreas in response to elevated circulating glucose levels. In homeostatic conditions, insulin induces lipogenesis to decrease plasma FFA. It also stimulates tissue glucose uptake by binding to the insulin receptors in adipose tissue and skeletal muscle, which activates glucose transporter GLUT4, thus lowering blood glucose levels. Under excessive caloric intake, the insulin demand increases and insulin receptor-expressing tissues become unresponsive to insulin as a protective mechanism to avoid excessive glucose uptake (162). As a result, pancreatic β-cells uncontrollably release insulin to overcome the desensitization, which eventually impairs β-cell function (35, 162). Insulin resistance is accompanied by adipose tissue inflammation, impaired adipokine release, downregulation of GLUT4, and ectopic fat accumulation resulting from increased FFA (52, 161, 163). Rodent studies have shown that high-fat diets contribute to insulin resistance by impairing peripheral glucose uptake and increasing pro-inflammatory cytokines gene expression in adipose tissue (52, 164). In humans, oral administration of palm oil decreased adipose tissue insulin sensitivity and increased hepatic fat deposition (165). Other clinical studies demonstrate that increased visceral fat deposition is significantly associated with insulin resistance, dyslipidemia, and hyperinsulinemia in healthy individuals (166).

Insulin is a pancreatic hormone that is crucial for glucose metabolism and energy balance. Insulin receptors are expressed in the hypothalamus, including the ARH and VMH (167, 168). In response to changes in circulating glucose, insulin is released from pancreatic β-cells. It enters the brain to directly act on ARH, blocking orexigenic effects of NPY/AgRP neurons and enhancing anorexigenic effects of POMC neurons to increase satiety and decrease blood glucose (167). Multiple studies have shown that OVX in rodents increases circulating insulin and glucose, decreases insulin sensitivity, and impairs β-cell function and insulin secretion. These phenotypes are exacerbated by HFD and accompanied by increased body weight and total body fat. Estrogen replacement in these animals lowers plasma insulin and glucose levels, increases glucokinase and GLUT2 expression, improves glucose tolerance and insulin sensitivity (169–172). Central estrogen signaling is suggested to be involved in insulin sensitivity, as intact obese female mice are protected from DIO-induced insulin resistance in NPY/AgRP neurons. Conversely, ovarian hormone depletion abolishes NPY/AgRP insulin sensitivity (117), and deletion of ERα in steroidogenic factor 1 (SF1) - positive neurons in the VMH impairs glucose tolerance in female mice (17, 20).

Insulin resistance may result not only from β-cell dysfunction or glucose disposal impairment but can also be caused by a reduction in insulin-mediated thermogenesis (173). BAT is highly sensitive to insulin given the high expression of insulin receptors in this tissue (35, 174). Insulin-mediated BAT activity improves lipid/glucose clearance, contributing to decreased glucolipotoxicity, ultimately improving β-cell function and insulin sensitivity (174). In humans, insulin stimulation increases BAT glucose uptake, energy expenditure, and expression of UCP1 and glucose transport genes in BAT (175). Similarly, cold exposure for eight hours increases energy expenditure, glucose disposal, and insulin sensitivity in individuals with high BAT activity. Administration of insulin following cold exposure further enhances glucose disposal and reduces glucose production in these patients (176). A study by Benedict et al. directly targeted central insulin signaling by administrating intranasal insulin to healthy men following an overnight fast. Individuals treated with insulin showed a dramatic increase in energy expenditure (177).

In mice, it has been shown that insulin acts through SNS to upregulate UCP1 in BAT and thermogenic function (178). In line with this, adrenergic stimulation in mice increases WAT lipolysis, BAT insulin signaling, and energy expenditure. These phenotypes are reversed by insulin receptor antagonism (179). Deletion of insulin receptors specifically in BAT results in decreases in glucose uptake, mass, and thermogenic capacity of BAT (179, 180). A recent report targeting diosmetin, a natural flavonoid commonly found in citrus fruits, showed that diosmetin treatment reverses DIO in mice by decreasing fat mass, reducing plasma glucose and insulin, and increasing insulin sensitivity and glucose disposal. In addition, diosmetin-treated mice show increased O_2_ consumption, enhanced heat production, and increased body temperature and WAT browning. Interestingly, mRNA levels of ERα and ERβ were dramatically increased in WAT and BAT, suggesting that diosmetin-induced thermogenesis was mediated by estrogen signaling. Administration of estrogen antagonist fulvestrant abolished the beneficial metabolic effects of diosmetin in DIO mice. These findings link insulin sensitivity and thermogenesis with estrogenic signals in adipose tissue.

### Mammalian Target of Rapamycin (mTOR)

Emerging evidence highlights the relevance of hypothalamic mTOR signaling in body weight balance and energy intake. mTOR is a serine-threonine kinase that senses fluctuations in nutrient availability (181, 182). In its phosphorylated (active, pmTOR) form, mTOR phosphorylates its major substrate, the serine/threonine ribosomal protein S6 kinase B1 (S6K1), modulating multiple cellular processes, including cell growth, cell survival, proliferation, and glucose metabolism (181, 182). Hypothalamic mTOR expression and activity have been reported in ARH, VMH, PVN, and the mediobasal hypothalamic area (MBH) (181–183).

In recent years, several reports have revealed the association between estrogen’s anorexic effects and mTOR signaling. An elegant study from González-García et al. provided evidence of mTOR-dependent weight loss and decreased food intake induced by E2. In addition to the typical obesogenic phenotypes associated with estradiol depletion, OVX rats showed reduced phosphorylation of mTOR and S6K1 in the ARH compared to sham rats. Conversely, E2 supplementation led to decreased body weight and food intake, associated with increased phosphorylation of mTOR and pS6K1 in the ARH. Consistently, ICV delivery of PPT reversed the OVX-induced body weight gain and strongly enhanced phosphorylation of mTOR and pS6K1 in the ARH (174). These findings support a model that E2/ERα activation increases mTOR signaling in the ARH to regulate food intake and body weight (182).

A recent report identified the role of the kappa-opioid receptor (k-OR) in the obesogenic effects of E2 withdrawal (175). They found that a k-OR/mTOR/p70S6K axis compensates for estrogens depletion in OVX mice by promoting body weight loss, increased energy expenditure, and WAT browning. Conversely, central antagonism of orexigenic k-OR increased phosphorylation of mTOR in the MBH of WT OVX mice, resulting in beneficial metabolic effects. Selective expression of constitutive activated S6K in the MBH decreased body weight and WAT mass and stimulated WAT browning, as indicated by increased UCP1 expression in gWAT. These findings suggest a model that inhibition of k-OR activates mTOR signaling in MBH to prevent body weight gain and adiposity induced by OVX (183).

## Ventromedial Hypothalamus (VMH)

The VMH plays a critical role in modulating energy expenditure, appetite regulation, sexual aggressive behaviors, lipid mobilization, and thermoregulation. The VMH was the first hypothalamic region identified to stimulate BAT thermogenesis and energy expenditure (19, 168). It is well established that VMH lesions in animals induce increases in food intake, adiposity, and body weight gain (184). Electrical stimulation of the VMH increases BAT temperature and thermogenic activity. This was abolished by the β-adrenergic blockade, confirming that SNS is an essential intermediary between the VMH-BAT crosstalk (185). SF1 is the most abundant neural population in the VMH. SF1 neurons express LepR, vesicular glutamate transporter 2 (vGLUT2), and ERα (19, 168). SF1 neurons project to the medial amygdala (MeA), DMH, periaqueductal gray (PAG), and ARH, all of which are linked to feeding regulation (186). Interestingly, recent reports have reveal that glutamatergic VMH neurons project to POMC neurons and that this VMH→POMC neural circuit senses changes in nutrient availability and helps maintain energy homeostasis (187). In addition to regulation of energy expenditure, the VMH neurons have been recently shown to be glucose-sensitive and play a key role in glucose homeostasis (188).

### Estrogen Signals in VMH Regulate Energy Balance

**Figure 2:**
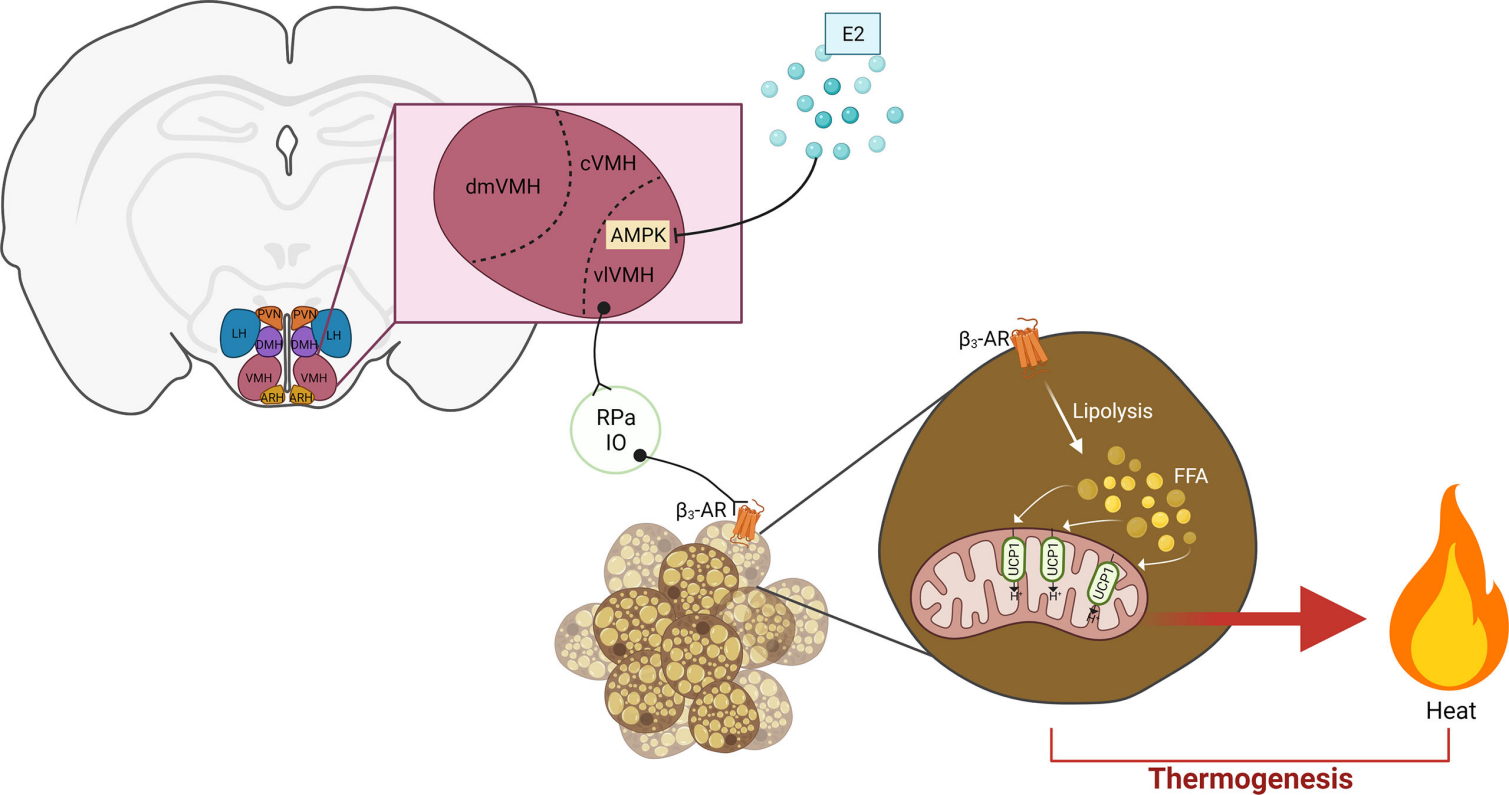
Hypothalamic estrogen acts on the VMH-AMPK-SNS-BAT axis to regulate thermogenesis. Estrogen represses AMPK signaling in the vlVMH to subsequently stimulate sympathetic activity. In the BAT, β3-AR activation induces lipolysis. The resulting FFA are oxidized in the mitochondria, providing the fuel for heat production by UCP1 activity. E2, estrogen; PVN, paraventricular hypothalamic nucleus; DMH, dorsomedial hypothalamus; LH, lateral hypothalamus; ARH, arcuate hypothalamic nucleus; VMH, ventromedial hypothalamus; dmVMH, dorsomedial VMH; cVMH, central VMH; vlVMH, ventrolateral CMH; IO, inferior olive; RPa, raphe pallidus; FFA, free fatty acid; UCP1, uncoupling protein 1. Adapted from (21), created by (194).

AMP-activated protein kinase (AMPK) is a critical element in the regulatory effects of VMH on BAT thermogenesis and glucose homeostasis. AMPK is activated by phosphorylation of Thr172 in the alpha subunit. Activation of AMPK signaling in the hypothalamus is associated with increased food intake and decreased energy expenditure (189). In the VMH, multiple factors have been found to repress AMPK activation and subsequently promote anti-obesogenic signals. These factors include bone morphogenetic protein 8B (BMP8B) (190), glucagon-like peptide 1 (GLP-1) (191), thyroid hormones (TH) (192), leptin (156), and estradiol (59). These AMPK-regulating factors have been extensively reviewed in a recent publication (193). The raphe pallidus (RPa) and inferior olive (IO) are two brainstem nuclei that are anatomically connected to the VMH and participate in the activation of SNS to activate BAT thermogenesis (**Figure 2**) (189).

Several studies have evidenced the requirement of VMH estrogen-ERα signaling for the modulation of BAT thermogenesis and fat distribution (16, 17, 19, 20, 57). Martínez de Morentin and colleagues unveiled the role of AMPK in the stimulatory effects of VMH estrogen signaling on BAT thermogenesis and energy expenditure (59). In their study, they showed that OVX rats treated with E2 directly into the VMH *via* ICV infusion show dramatic loss of body weight gain and food intake, increases mRNA levels and protein content of UCP1 in BAT and increases BAT temperature. All these effects were associated with decreased AMPK phosphorylation (active) in the VMH, suggesting a protentional role of VMH AMPK signaling. In supporting this, specific injection of E2 into the VMH decreased VMH AMPK activity, increased c-fos numbers in RPa and IO, and upregulated expression of thermogenic markers in the BAT. Conversely, VMH-specific co-infusion of AMPK completely abolished these anti-obesity effects (59). These findings reveal a VMH-AMPK-SNS-BAT axis that is modulated by estrogenic signals to maintain energy balance (**Figure 2**). Other studies have shown that silencing ERα in the VMH of rodents induces body weight gain, increased visceral adiposity, decreased physical activity, downregulation of thermogenic genes in the BAT, and increased circulating glucose (16, 17). In a study performed by Xu and colleagues, female mice with ERα deleted specifically in the SF1 neurons demonstrate increased body weight, increased visceral and gonadal adiposity, impaired glucose tolerance, decreased heat production, as well as decreased SNS activity and BAT UCP1 transcripts. The group concluded that estrogens activate ERα expressed by the SF1 neurons of the VMH to increase sympathetic outflow and modulate fat distribution, which ultimately results in increased BAT thermogenesis and decreased lipid storage (20).

Besides the VMH-AMPK-SNS-BAT axis, E2 also modulates ceramides metabolism and endoplasmic reticulum (ER) stress in the brain to regulate thermogenesis and energy balance (187). Specifically, OVX increased levels of ER stress markers in MBH and VMH, while both E2 replenishment and ICV ceramide inhibition abolished these phenotypes in rats. This group further targeted serine palmitoyltransferase long chain base subunit 1 (SPTLC1), an enzyme that catalyzes ceramide synthesis, to explore ceramide action in the VMH. They found that VMH-specific injection of SPTLC1 shRNA decreased body weight, enhanced BAT and core temperature, and upregulated BAT UCP1 protein levels in OVX rats. These phenotypes were accompanied by the downregulation of ER stress markers in the VMH. Consistently, VMH-specific overexpression of ER glucose chaperone GPR78, a protein that decreases ER stress by facilitating degradation pathways, also ameliorated ER stress and improved body weight and thermoregulation in OVX rats. These findings demonstrate that estrogens in the VMH contribute to energy balance by maintaining optimal levels of ceramides and decreasing ER stress (187).

ERα is highly expressed in the ventrolateral subregion of the VMH (vlVMH), where it has been shown to regulate fertility, social behaviors, and locomotor activity in a sex-specific manner (16, 18–20, 99). We and others have shown that DREADD activation of ERα^vlVMH^ neurons augmented BAT and core temperature as well as physical activity in female mice (18, 195). Moreover, this ERα^vlVMH^ chemogenetic activation ameliorates body weight gain caused by HFD, indicating beneficial effects of ERα^vlVMH^ activation in metabolic health (195). Correa et al. targeted Nkx2-1^+^ neurons in the vlVMH, which are associated with physical activity in mice. ERα and NKX2-1 are co-expressed in the vlVMH. Neural activation of Nkx2-1^+^ vlVMH using chemogenetic approaches increased ambulatory and vertical movement as well as heat production in Nkx2-1Cre female mice. Deletion of NKX2-1 in the vlVMH of female mice led to increased body weight, increased WAT depots, decreased locomotion, and lower heat production. More importantly, these phenotypes were associated with decreased *Esr1* transcripts and ERα protein content (19). A single-cell transcriptomic study targeting SF1^+^ neurons in the VMH identified differentially expressed gene transcripts that included reprimo (*Rprm*), tachykinin Precursor 1 (*Tac1*), prodynorphin (*Pdyn*), and somatostatin (*Sst*). In females, *Tac1* and *Rprm* were highly expressed in the vlVMH when compared to males, and these genes were found to coexpress with ERα in the vlVMH (ERα^vlVMH^), positioning these genes as female-specific markers in this neuronal population (16, 18).

A recent report has identified another sexually dimorphic subset of ERα^vlVMH^ neurons expressing MC4R, a receptor of anorexigenic ligand melanocyte-stimulating hormones (MSH) (16, 196). Loss-of-function mutations in *MC4R* are the most frequent genetic cause of obesity in humans (196). In this study, ERα/MC4R^vlVMH^ neurons were identified as a subset population of ERα^vlVMH^ neurons. They showed colocalization of MC4R and ERα in the vlVMH during proestrus in mice supplemented with E2. Although OVX mice display reduced locomotion and hypometabolism, chemogenetic manipulation of ERα/MC4R^vlVMH^ neurons significantly stimulated physical activity in these estrogen-depleted mice. The restoration of physical activity by ERα/MC4R^vlVMH^ stimulation was accompanied by reduced body weight and white adipocyte size. These findings suggest that ERα/MC4R^vlVMH^ plays an essential role in sex-dimorphic physical activity in mice (16).

To gain mechanistic insight into the physiological functions of ERα^vlVMH^ neural subsets, the anatomical distribution of the downstream neural circuitries and their functionality need to be studied. Excitingly, MC4R^vlVMH^ neurons were shown to send projections to distinct brain regions including dorsal CA1, periaqueductal grey (PAG), and hindbrain pontine region (16). Similarly, we have recently identified 5-HT^+^ neurons in the DRN as a downstream neuronal population receiving monosynaptic innervation from ERα^vlVMH^. Using electrophysiology and optogenetic tools, we showed the glutamatergic nature of these ERα^vlVMH^ → DRN monosynaptic inputs. Interestingly, we demonstrate that ERα^vlVMH^ → DRN neural circuit has thermo-sensing and nutrient-sensing properties, which are abolished in OVX mice, suggesting an important role of ovarian hormones in ERα^vlVMH^ → DRN responsiveness to ambient temperature and nutritional state. Optogenetic activation of the ERα^vlVMH^ → DRN circuit stimulates BAT thermogenesis and physical activity, while its inhibition has the opposite effect, demonstrating an *in vivo* physiological role ERα^vlVMH^ → DRN neural circuit. We show that activation of 5-HT^DRN^ neurons is required for these stimulatory effects induced by ERα^vlVMH^ activity. Importantly, deletion of ERα from vlVMH neurons projecting to DRN reduced physical activity and BAT thermogenesis in female mice, demonstrating that ERα^vlVMH^ → DRN neural circuit is relevant for regulation of physical activity and energy expenditure in female mice (195). Altogether, these findings demonstrate the physiological relevance of ERα^vlVMH^ neurons in the maintenance of physical activity and thermogenic balance.

As discussed previously, the VMH communicates with both BAT and WAT through SNS to modulate thermogenesis and lipolysis. The VMH regulation of BAT thermogenesis has been discussed. However, VMH modulation of WAT lipolysis is less clear. Like BAT’s thermogenesis mechanism, SNS induces the release of NE, which activates β3-adrenoceptors in white adipocytes. The subsequent downstream signaling cascade stimulates PKA, which phosphorylates perilipin A and HSL, two key factors that initiate lipolysis (197). One of the possible contributors of impaired SNS activation during obesity is insulin. It is suggested that insulin resistance may increase sympathetic firing and therefore increase lipolytic rate in WAT, which results in elevated free fatty acids in circulation (197). The VMH-AMPK-SNS-BAT axis has been discussed before. However, despite the evidence indicating the role of estrogen VMH signaling in adiposity and fat distribution, the neuroanatomical projections that mediate the estrogen VMH-SNS-WAT communication, as well as the underlying molecular mechanisms, remain to be elucidated.

### Estrogen Signals in the VMH Regulate Glucose Homeostasis

In the brain, two different types of neurons can sense changes in circulating glucose levels: glucose-excited (GE) neurons and glucose-inhibited (GI) neurons (198). The VMH is highly enriched with both GE and GI neurons. Within the vlVMH, GE neurons are more abundant than GI. Additionally, the majority of ERα^vlVMH^ neurons are glucose-sensing neurons (188). The enzyme glucokinase is expressed in GE and GI neurons. It is required for the glucose-sensing property, as ablation of this enzyme in the VMH leads to increased fat mass, decreased glucagon secretion, and reduced sympathetic activity. These phenotypes were predominant in female mice, highlighting the already mentioned sexual-biased functions in the vlVMH (199). In GE neurons, when glucose levels are elevated, the ATP/ADP ratio is increased. This, in turn, causes the closure of K_ATP_ channels, resulting in the depolarization and excitation of these neurons. In GI neurons, the increased glucose also increases the ATP/ADP ratio, but this results in the inhibition of AMPK. As a result, the K^+^ and Cl^-^ channels are opened, allowing the hyperpolarization and inhibition of GI neurons (198, 200). These mechanisms ultimately decrease the levels of glucose in circulation. Under low glucose conditions, K_ATP_ channels are opened, and GE neurons are hyperpolarized. GI neurons are depolarized by activation of AMPK, which induces closure of K^+^ and Cl^-^ channels. Inhibition of GE neurons and activation of GI neurons leads to an increase in circulating glucagon, which ultimately results in increased glucose levels (188, 200).

Since estrogen is a key factor in the VMH-AMPK-SNS-BAT axis, it is not surprising that under hypoglycemic conditions, estradiol impedes the activation of GI neurons in female mice by inhibiting AMPK signaling, thus preventing the decrease in glucose concentrations in a sex-specific manner (201). Yu et al. demonstrated the requirement of membrane-bound ERα for fasting- and glucopenia-induced refeeding. Mutations in membrane-bound ERα decreased the firing rate of ERα^vlVMH^ even after administration of PPT, and importantly, impaired the ability of ERα^vlVMH^ neurons to respond to hypoglycemia (101). Our previous study reveals the mechanisms underlying the glucose-sensing properties in ERα^vlVMH^ neurons (188). During hypoglycemia, the activation of GI neurons in ERα^vlVMH^ neurons is mediated by the calcium-activated chloride channel protein channel encoded by anoctamin 4 (*Ano4*), which contributes to their depolarization. Conversely, GE neurons are hyperpolarized by the activation of *Abcc8*, which encodes K_ATP_ subunit Sur1. Additionally, we reported that GI-ERα^vlVMH^ neurons project to the medioposterior part of the ARH (mpARH) and GE-ERα^vlVMH^ neurons project to the DRN. Activation of ERα^vlVMH^→mpARH and inhibition of ERα^vlVMH^→DRN complementarily contribute to the prevention of hypoglycemia (188). Altogether, the discussed evidence supports estrogen as a critical element in the VMH for the modulation of glucose and energy homeostasis.

## Conclusion

Obesity is considered a global epidemic affecting 650 million people worldwide (128, 202). This disease is often exacerbated by other metabolic disorders that have a significant impact on patient quality of life. In humans and animal models, sexual dimorphism has been described not only in adipose tissue deposition and distribution but also in BAT thermogenic capacity. This is partially mediated by the female sex hormone estrogen, which confers protection against metabolic diseases in premenopausal women. Hypothalamic estrogen signaling has been shown to have positive effects on energy homeostasis by decreasing food consumption, diminishing excessive adiposity, improving insulin sensitivity, and promoting BAT thermogenesis and WAT beiging. Although it is well established that SNS mediates estrogen brain-to-adipose tissue signals (57, 59), less is known about the downstream neural circuits mediating the regulatory effects of brain estrogen on adipose metabolism. As discussed earlier, multiple hypothalamic projections have been described, including the VMH→POMC neural circuit that detects fluctuations in nutritional status and the ERα^vlVMH^ → DRN that modulates physical activity and energy expenditure (187, 195). It would be interesting to test the role of estrogen in VMH→POMC projection and explore in more depth the estrogen-modulated hypothalamic circuitry that regulates adipose tissue function and remodeling. Moreover, while ERα^+^ neurons in the VMH have been shown to be essential for heat production and energy expenditure (20), a recent report also revealed that ERα^+^ neurons in the MPOA are thermosensitive and decrease energy expenditure when activated (136). These findings support a model where ERα^+^ neurons in distinct hypothalamic regions act synergistically to respond to changes in nutrient availability and environmental temperature. Collectively, further studies are needed to unravel the estrogen-governed neural networks modulating adipose tissue function, which is critical for a better understanding of the pathophysiology of obesity.

## Author Contributions

VTI is the main contributor to the manuscript writing; YJ, YH, and PX contributed to the manuscript design, discussion and revision. All authors contributed to the article and approved the submitted version.

## Funding

The work in our laboratories was partially supported by grants from NIH (R01 DK123098 to PX; P20 GM135002, R01 DK129548 to YH; R03 DK127149 to YJ; T32 AA026577 to VI), DOD (Innovative Grant W81XWH-19-PRMRP-DA to PX), and DRTC (The Pilot and Feasibility Award DK020595 to PX).

## Conflict of Interest

The authors declare that the research was conducted in the absence of any commercial or financial relationships that could be construed as a potential conflict of interest.

## Publisher’s Note

All claims expressed in this article are solely those of the authors and do not necessarily represent those of their affiliated organizations, or those of the publisher, the editors and the reviewers. Any product that may be evaluated in this article, or claim that may be made by its manufacturer, is not guaranteed or endorsed by the publisher.

## References

[1] World Health Organ. Obesity. Available at: https://www.who.int/westernpacific/health-topics/obesity (Accessed January 28, 2022).

[2] Ruiz JR Martinez-Tellez B Sanchez-Delgado G Osuna-Prieto FJ Rensen PCN Boon MR. Role of Human Brown Fat in Obesity, Metabolism and Cardiovascular Disease: Strategies to Turn Up the Heat. Prog Cardiovasc Dis (2018) 61:232–45. doi: 10.1016/j.pcad.2018.07.002 29981351

[3] Longo M Zatterale F Naderi J Parrillo L Formisano P Raciti GA. Adipose Tissue Dysfunction as Determinant of Obesity-Associated Metabolic Complications. Int J Mol Sci (2019) 20:E2358. doi: 10.3390/ijms20092358 31085992 PMC6539070

[4] Fuster JJ Ouchi N Gokce N Walsh K. Obesity-Induced Changes in Adipose Tissue Microenvironment and Their Impact on Cardiovascular Disease. Circ Res (2016) 118:1786–807. doi: 10.1161/CIRCRESAHA.115.306885 PMC4887147 27230642

[5] Legato MJ. Gender-Specific Aspects of Obesity. Int J Fertil Womens Med (1997) 42:184–97. 9222803

[6] Canoy D. Distribution of Body Fat and Risk of Coronary Heart Disease in Men and Women. Curr Opin Cardiol (2008) 23:591–8. doi: 10.1097/HCO.0b013e328313133a 18830075

[7] Pi-Sunyer FX. The Epidemiology of Central Fat Distribution in Relation to Disease. Nutr Rev (2004) 62:S120–6. doi: 10.1111/j.1753-4887.2004.tb00081.x 15387477

[8] Manolopoulos KN Karpe F Frayn KN. Gluteofemoral Body Fat as a Determinant of Metabolic Health. Int J Obes (2010) 34:949–59. doi: 10.1038/ijo.2009.286 20065965

[9] Cypess AM Lehman S Williams G Tal I Rodman D Goldfine AB. Identification and Importance of Brown Adipose Tissue in Adult Humans. N Engl J Med (2009) 360:1509–17. doi: 10.1056/NEJMoa0810780 PMC2859951 19357406

[10] Hoyenga KB Hoyenga KT. Gender and Energy Balance: Sex Differences in Adaptations for Feast and Famine. Physiol Behav (1982) 28:545–63. doi: 10.1016/0031-9384(82)90153-6 7043508

[11] Cooper AJ Gupta SR Moustafa AF Chao AM. Sex/Gender Differences in Obesity Prevalence, Comorbidities, and Treatment. Curr Obes Rep (2021) 10:458–66. doi: 10.1007/s13679-021-00453-x 34599745

[12] D’Eon TM Souza SC Aronovitz M Obin MS Fried SK Greenberg AS. Estrogen Regulation of Adiposity and Fuel Partitioning: Evidence Of Genomic And Non-Genomic Regulation Of Lipogenic And Oxidative Pathways *. J Biol Chem (2005) 280:35983–91. doi: 10.1074/jbc.M507339200 16109719

[13] Palmer K Gray JM. Central vs. Peripheral Effects of Estrogen on Food Intake and Lipoprotein Lipase Activity in Ovariectomized Rats. Physiol Behav (1986) 37:187–9. doi: 10.1016/0031-9384(86)90404-X 3737718

[14] Benz V Bloch M Wardat S Böhm C Maurer L Mahmoodzadeh S. Sexual Dimorphic Regulation of Body Weight Dynamics and Adipose Tissue Lipolysis. PloS One (2012) 7:e37794. doi: 10.1371/journal.pone.0037794 22662224 PMC3360591

[15] Zhu L Yang Y Xu P Zou F Yan X Liao L. Steroid Receptor Coactivator-1 Mediates Estrogenic Actions to Prevent Body Weight Gain in Female Mice. Endocrinology (2013) 154:150–8. doi: 10.1210/en.2012-2007 PMC3529365 23211707

[16] Krause WC Rodriguez R Gegenhuber B Matharu N Rodriguez AN Padilla-Roger AM. Oestrogen Engages Brain MC4R Signalling to Drive Physical Activity in Female Mice. Nature (2021) 599:131–5. doi: 10.1038/s41586-021-04010-3 PMC9113400 34646010

[17] Musatov S Chen W Pfaff DW Mobbs CV Yang X-J Clegg DJ. Silencing of Estrogen Receptor Alpha in the Ventromedial Nucleus of Hypothalamus Leads to Metabolic Syndrome. Proc Natl Acad Sci USA (2007) 104:2501–6. doi: 10.1073/pnas.0610787104 PMC1892990 17284595

[18] van Veen JE Kammel LG Bunda PC Shum M Reid MS Massa MG. Hypothalamic Estrogen Receptor Alpha Establishes a Sexually Dimorphic Regulatory Node of Energy Expenditure. Nat Metab (2020) 2:351–63. doi: 10.1038/s42255-020-0189-6 PMC7202561 32377634

[19] Correa SM Newstrom DW Warne JP Flandin P Cheung CC Lin-Moore AT. An Estrogen-Responsive Module in the Ventromedial Hypothalamus Selectively Drives Sex-Specific Activity in Females. Cell Rep (2015) 10:62–74. doi: 10.1016/j.celrep.2014.12.011 25543145 PMC4324838

[20] Xu Y Nedungadi TP Zhu L Sobhani N Irani BG Davis KE. Distinct Hypothalamic Neurons Mediate Estrogenic Effects on Energy Homeostasis and Reproduction. Cell Metab (2011) 14:453–65. doi: 10.1016/j.cmet.2011.08.009 PMC3235745 21982706

[21] Xu Y López M. Central Regulation of Energy Metabolism by Estrogens. Mol Metab (2018) 15:104–15. doi: 10.1016/j.molmet.2018.05.012 PMC6066788 29886181

[22] Kim JH Cho HT Kim YJ. The Role of Estrogen in Adipose Tissue Metabolism: Insights Into Glucose Homeostasis Regulation [Review]. Endocr J (2014) 61:1055–67. doi: 10.1507/endocrj.EJ14-0262 25109846

[23] NHLBI. Clinical Guidelines on the Identification, Evaluation, and Treatment of Overweight and Obesity in Adults: The Evidence Report (1998). National Heart, Lung, and Blood Institute. Available at: https://www.ncbi.nlm.nih.gov/books/NBK1995/ (Accessed October 13, 2021).

[24] Panuganti KK Nguyen M Kshirsagar RK. “Obesity” StatPearls, in: Treasure Island (2021). StatPearls Publishing. Available at: http://www.ncbi.nlm.nih.gov/books/NBK459357/ (Accessed October 13, 2021).

[25] Magkos F Fraterrigo G Yoshino J Luecking C Kirbach K Kelly SC. Effects of Moderate and Subsequent Progressive Weight Loss on Metabolic Function and Adipose Tissue Biology in Humans With Obesity. Cell Metab (2016) 23:591–601. doi: 10.1016/j.cmet.2016.02.005 26916363 PMC4833627

[26] Shai I Schwarzfuchs D Henkin Y Shahar DR Witkow S Greenberg I. Weight Loss With a Low-Carbohydrate, Mediterranean, or Low-Fat Diet. N Engl J Med (2009) 359:229–41. doi: 10.1056/NEJMoa0708681 18635428

[27] Villareal DT Aguirre L Gurney AB Waters DL Sinacore DR Colombo E. Aerobic or Resistance Exercise, or Both, in Dieting Obese Older Adults. N Engl J Med (2017) 359:1943–55. doi: 10.1056/NEJMoa1616338 PMC5552187 28514618

[28] Cheng M Mei B Zhou Q Zhang M Huang H Han L. Computational Analyses of Obesity Associated Loci Generated by Genome-Wide Association Studies. PloS One (2018) 13:e0199987. doi: 10.1371/journal.pone.0199987 29966015 PMC6028139

[29] Dubern B Lubrano-Berthelier C Mencarelli M Ersoy B Frelut M-L Bouglé D. Mutational Analysis of the Pro-Opiomelanocortin Gene in French Obese Children Led to the Identification of a Novel Deleterious Heterozygous Mutation Located in the α-Melanocyte Stimulating Hormone Domain. Pediatr Res (2008) 63:211–6. doi: 10.1203/PDR.0b013e31815ed62b 18091355

[30] Farooqi IS Drop S Clements A Keogh JM Biernacka J Lowenbein S. Heterozygosity for a POMC-Null Mutation and Increased Obesity Risk in Humans. Diabetes (2006) 55:2549–53. doi: 10.2337/db06-0214 16936203

[31] Farooqi IS Keogh JM Yeo GSH Lank EJ Cheetham T O’Rahilly S. Clinical Spectrum of Obesity and Mutations in the Melanocortin 4 Receptor Gene. N Engl J Med (2003) 348:1085–95. doi: 10.1056/NEJMoa022050 12646665

[32] Lawler K Huang-Doran I Sonoyama T Collet T-H Keogh JM Henning E. Leptin-Mediated Changes in the Human Metabolome. J Clin Endocrinol Metab (2020) 105:2541–52. doi: 10.1210/clinem/dgaa251 PMC7282709 32392278

[33] Mendiratta MS Yang Y Balazs AE Willis AS Eng CM Karaviti LP. Early Onset Obesity and Adrenal Insufficiency Associated With a Homozygous POMC Mutation. Int J Pediatr Endocrinol (2011) 2011:5. doi: 10.1186/1687-9856-2011-5 21860632 PMC3159139

[34] Montague CT Farooqi IS Whitehead JP Soos MA Rau H Wareham NJ. Congenital Leptin Deficiency Is Associated With Severe Early-Onset Obesity in Humans. Nature (1997) 387:903–8. doi: 10.1038/43185 9202122

[35] Chait A den Hartigh LJ. Adipose Tissue Distribution, Inflammation and Its Metabolic Consequences, Including Diabetes and Cardiovascular Disease. Front Cardiovasc Med (2020) 7:22. doi: 10.3389/fcvm.2020.00022 32158768 PMC7052117

[36] BioRender.com (Toronto, CA). Adipose Tissue Depots. (2022) Available at: app.biorender.com/biorender-templates.

[37] Langin D Dicker A Tavernier G Hoffstedt J Mairal A Rydén M. Adipocyte Lipases and Defect of Lipolysis in Human Obesity. Diabetes (2005) 54:3190–7. doi: 10.2337/diabetes.54.11.3190 16249444

[38] Chandran M Phillips SA Ciaraldi T Henry RR. Adiponectin: More Than Just Another Fat Cell Hormone? Diabetes Care (2003) 26:2442–50. doi: 10.2337/diacare.26.8.2442 12882876

[39] Harmelen VV Reynisdottir S Eriksson P Thörne A Hoffstedt J Lönnqvist F. Leptin Secretion From Subcutaneous and Visceral Adipose Tissue in Women. Diabetes (1998) 47:913–7. doi: 10.2337/diabetes.47.6.913 9604868

[40] Li Y Jiang C Xu G Wang N Zhu Y Tang C. Homocysteine Upregulates Resistin Production From Adipocytes *In Vivo* and *In Vitro* . Diabetes (2008) 57:817–27. doi: 10.2337/db07-0617 18192543

[41] Zhang Y Proenca R Maffei M Barone M Leopold L Friedman JM. Positional Cloning of the Mouse Obese Gene and its Human Homologue. Nature (1994) 372:425–32. doi: 10.1038/372425a0 7984236

[42] Hou B Zhao Y He P Xu C Ma P Lam SM. Targeted Lipidomics and Transcriptomics Profiling Reveal the Heterogeneity of Visceral and Subcutaneous White Adipose Tissue. Life Sci (2020) 245:117352. doi: 10.1016/j.lfs.2020.117352 32006527 PMC7988272

[43] Ross R Shaw KD Martel Y de Guise J Hudson R Avruch L. “Determination of Total and Regional Adipose Tissue Distribution by Magnetic Resonance Imaging in Android Women.,”. In: Ellis KJ Eastman JD, editors. Human Body Composition: In Vivo Methods, Models, and Assessment. Basic Life Sciences. Boston, MA: Springer US (1993). p. 177–80. doi: 10.1007/978-1-4899-1268-8_40 8110104

[44] Stinkens R Brouwers B Jocken JW Blaak EE Teunissen-Beekman KF Hesselink MK. Exercise Training-Induced Effects on the Abdominal Subcutaneous Adipose Tissue Phenotype in Humans With Obesity. J Appl Physiol (2018) 125:1585–93. doi: 10.1152/japplphysiol.00496.2018 30212302

[45] Fontana L Eagon JC Trujillo ME Scherer PE Klein S. Visceral Fat Adipokine Secretion Is Associated With Systemic Inflammation in Obese Humans. Diabetes (2007) 56:1010–3. doi: 10.2337/db06-1656 17287468

[46] Liu L Feng J Zhang G Yuan X Li F Yang T. Visceral Adipose Tissue is More Strongly Associated With Insulin Resistance Than Subcutaneous Adipose Tissue in Chinese Subjects With Pre-Diabetes. Curr Med Res Opin (2018) 34:123–9. doi: 10.1080/03007995.2017.1364226 28776439

[47] Matsha TE Ismail S Speelman A Hon GM Davids S Erasmus RT. Visceral and Subcutaneous Adipose Tissue Association With Metabolic Syndrome and its Components in a South African Population. Clin Nutr ESPEN (2019) 32:76–81. doi: 10.1016/j.clnesp.2019.04.010 31221294

[48] Jo J Gavrilova O Pack S Jou W Mullen S Sumner AE. Hypertrophy and/or Hyperplasia: Dynamics of Adipose Tissue Growth. PloS Comput Biol (2009) 5:e1000324. doi: 10.1371/journal.pcbi.1000324 19325873 PMC2653640

[49] Muir LA Neeley CK Meyer KA Baker NA Brosius AM Washabaugh AR. Adipose Tissue Fibrosis, Hypertrophy, and Hyperplasia: Correlations With Diabetes in Human Obesity. Obesity (2016) 24:597–605. doi: 10.1002/oby.21377 26916240 PMC4920141

[50] Spalding KL Bernard S Näslund E Salehpour M Possnert G Appelsved L. Impact of Fat Mass and Distribution on Lipid Turnover in Human Adipose Tissue. Nat Commun (2017) 8:15253. doi: 10.1038/ncomms15253 28534500 PMC5457499

[51] Unamuno X Gómez-Ambrosi J Rodríguez A Becerril S Frühbeck G Catalán V. Adipokine Dysregulation and Adipose Tissue Inflammation in Human Obesity. Eur J Clin Invest (2018) 48:e12997. doi: 10.1111/eci.12997 29995306

[52] van der Heijden RA Sheedfar F Morrison MC Hommelberg PP Kor D Kloosterhuis NJ. High-Fat Diet Induced Obesity Primes Inflammation in Adipose Tissue Prior to Liver in C57BL/6j Mice. Aging (2015) 7:256–68. doi: 10.18632/aging.100738 PMC4429090 25979814

[53] Chitraju C Fischer AW Farese RV Walther TC. Lipid Droplets in Brown Adipose Tissue Are Dispensable for Cold-Induced Thermogenesis. Cell Rep (2020) 33:108348. doi: 10.1016/j.celrep.2020.108348 33147469 PMC7696656

[54] Darcy J Wang C-H Tseng Y-H. “Analyzing Mitochondrial Function in Brown Adipocytes With a Bioenergetic Analyzer”. In: Clinical and Preclinical Models for Maximizing Healthspan: Methods and Protocols. Methods in Molecular Biology. New York, NY: Springer US (2020). p. 289–96. doi: 10.1007/978-1-0716-0471-7_20 PMC8075131 32219757

[55] Milton-Laskíbar I Gómez-Zorita S Arias N Romo-Miguel N González M Fernández-Quintela A. Effects of Resveratrol and its Derivative Pterostilbene on Brown Adipose Tissue Thermogenic Activation and on White Adipose Tissue Browning Process. J Physiol Biochem (2020) 76:269–78. doi: 10.1007/s13105-020-00735-3 32170654

[56] Saito M Okamatsu-Ogura Y Matsushita M Watanabe K Yoneshiro T Nio-Kobayashi J. High Incidence of Metabolically Active Brown Adipose Tissue in Healthy Adult Humans: Effects of Cold Exposure and Adiposity. Diabetes (2009) 58:1526–31. doi: 10.2337/db09-0530 PMC2699872 19401428

[57] Kim S-N Jung Y-S Kwon H-J Seong JK Granneman JG Lee Y-H. Sex Differences in Sympathetic Innervation and Browning of White Adipose Tissue of Mice. Biol Sex Differ (2016) 7:67. doi: 10.1186/s13293-016-0121-7 27990249 PMC5148917

[58] Lim S Honek J Xue Y Seki T Cao Z Andersson P. Cold-Induced Activation of Brown Adipose Tissue and Adipose Angiogenesis in Mice. Nat Protoc (2012) 7:606–15. doi: 10.1038/nprot.2012.013 22383039

[59] Martínez de Morentin PB González-García I Martins L Lage R Fernández-Mallo D Martínez-Sánchez N. Estradiol Regulates Brown Adipose Tissue Thermogenesis *via* Hypothalamic AMPK. Cell Metab (2014) 20:41–53. doi: 10.1016/j.cmet.2014.03.031 24856932 PMC4082097

[60] Bo S Fadda M Castiglione A Ciccone G De Francesco A Fedele D. Is the Timing of Caloric Intake Associated With Variation in Diet-Induced Thermogenesis and in the Metabolic Pattern? A Randomized Cross-Over Study. Int J Obes (2015) 39:1689–95. doi: 10.1038/ijo.2015.138 26219416

[61] Chen KY Brychta RJ Linderman JD Smith S Courville A Dieckmann W. Brown Fat Activation Mediates Cold-Induced Thermogenesis in Adult Humans in Response to a Mild Decrease in Ambient Temperature. J Clin Endocrinol Metab (2013) 98:E1218–1223. doi: 10.1210/jc.2012-4213 PMC3701264 23780370

[62] Leitner BP Weiner LS Desir M Kahn PA Selen DJ Tsang C. Kinetics of Human Brown Adipose Tissue Activation and Deactivation. Int J Obes (2019) 43:633–7. doi: 10.1038/s41366-018-0104-3 PMC6252171 29795459

[63] Murano I Barbatelli G Giordano A Cinti S. Noradrenergic Parenchymal Nerve Fiber Branching After Cold Acclimatisation Correlates With Brown Adipocyte Density in Mouse Adipose Organ. J Anat (2009) 214:171–8. doi: 10.1111/j.1469-7580.2008.01001.x PMC2667925 19018882

[64] Fujikawa T Choi Y-H Yang DJ Shin DM Donato J Kohno D. P110β in the Ventromedial Hypothalamus Regulates Glucose and Energy Metabolism. Exp Mol Med (2019) 51:1–9. doi: 10.1038/s12276-019-0249-8 PMC6486607 31028248

[65] Fisher ffolliott M Kleiner S Douris N Fox EC Mepani RJ Verdeguer F. FGF21 Regulates PGC-1α and Browning of White Adipose Tissues in Adaptive Thermogenesis. Genes Dev (2012) 26:271–81. doi: 10.1101/gad.177857.111 PMC3278894 22302939

[66] Stanford KI Middelbeek RJW Townsend KL An D Nygaard EB Hitchcox KM. Brown Adipose Tissue Regulates Glucose Homeostasis and Insulin Sensitivity. J Clin Invest (2013) 123:215–23. doi: 10.1172/JCI62308 PMC3533266 23221344

[67] Olsen JM Sato M Dallner OS Sandström AL Pisani DF Chambard J-C. Glucose Uptake in Brown Fat Cells is Dependent on mTOR Complex 2–Promoted GLUT1 Translocation. J Cell Biol (2014) 207:365–74. doi: 10.1083/jcb.201403080 PMC4226734 25385184

[68] Hanssen MJW Wierts R Hoeks J Gemmink A Brans B Mottaghy FM. Glucose Uptake in Human Brown Adipose Tissue Is Impaired Upon Fasting-Induced Insulin Resistance. Diabetologia (2015) 58:586–95. doi: 10.1007/s00125-014-3465-8 25500952

[69] Ikeda K Maretich P Kajimura S. The Common and Distinct Features of Brown and Beige Adipocytes. Trends Endocrinol Metab (2018) 29:191–200. doi: 10.1016/j.tem.2018.01.001 29366777 PMC5826798

[70] Shabalina IG Petrovic N de Jong JMA Kalinovich AV Cannon B Nedergaard J. UCP1 in Brite/Beige Adipose Tissue Mitochondria Is Functionally Thermogenic. Cell Rep (2013) 5:1196–203. doi: 10.1016/j.celrep.2013.10.044 24290753

[71] Finlin BS Memetimin H Confides AL Kasza I Zhu B Vekaria HJ. Human Adipose Beiging in Response to Cold and Mirabegron. JCI Insight (2021) 3:e121510. doi: 10.1172/jci.insight.121510 PMC6129119 30089732

[72] Wang Q Zhang M Xu M Gu W Xi Y Qi L. Brown Adipose Tissue Activation Is Inversely Related to Central Obesity and Metabolic Parameters in Adult Human. PloS One (2015) 10:e0123795. doi: 10.1371/journal.pone.0123795 25894250 PMC4403996

[73] Yoneshiro T Aita S Matsushita M Okamatsu-Ogura Y Kameya T Kawai Y. Age-Related Decrease in Cold-Activated Brown Adipose Tissue and Accumulation of Body Fat in Healthy Humans. Obesity (2011) 19:1755–60. doi: 10.1038/oby.2011.125 21566561

[74] Matsushita M Yoneshiro T Aita S Kameya T Sugie H Saito M. Impact of Brown Adipose Tissue on Body Fatness and Glucose Metabolism in Healthy Humans. Int J Obes (2014) 38:812–7. doi: 10.1038/ijo.2013.206 24213309

[75] Feldmann HM Golozoubova V Cannon B Nedergaard J. UCP1 Ablation Induces Obesity and Abolishes Diet-Induced Thermogenesis in Mice Exempt From Thermal Stress by Living at Thermoneutrality. Cell Metab (2009) 9:203–9. doi: 10.1016/j.cmet.2008.12.014 19187776

[76] Yoneshiro T Aita S Matsushita M Kayahara T Kameya T Kawai Y. Recruited Brown Adipose Tissue as an Antiobesity Agent in Humans. J Clin Invest (2013) 123:3404. doi: 10.1172/JCI67803 23867622 PMC3726164

[77] Cypess AM Weiner LS Roberts-Toler C Elía EF Kessler SH Kahn PA. Activation of Human Brown Adipose Tissue by a β3-Adrenergic Receptor Agonist. Cell Metab (2015) 21:33–8. doi: 10.1016/j.cmet.2014.12.009 PMC4298351 25565203

[78] O’Mara AE Johnson JW Linderman JD Brychta RJ McGehee S Fletcher LA. Chronic Mirabegron Treatment Increases Human Brown Fat, HDL Cholesterol, and Insulin Sensitivity. J Clin Invest (2020) 130:2209–19. doi: 10.1172/JCI131126 PMC7190915 31961826

[79] Lemieux S Prud’homme D Bouchard C Tremblay A Després JP. Sex Differences in the Relation of Visceral Adipose Tissue Accumulation to Total Body Fatness. Am J Clin Nutr (1993) 58:463–7. doi: 10.1093/ajcn/58.4.463 8379501

[80] Porter JW Barnas JL Welly R Spencer N Pitt J Vieira-Potter VJ. Age, Sex, and Depot-Specific Differences in Adipose-Tissue Estrogen Receptors in Individuals With Obesity. Obes Silver Spring Md (2020) 28:1698–707. doi: 10.1002/oby.22888 PMC7483923 32734695

[81] Grove KL Fried SK Greenberg AS Xiao XQ Clegg DJ. A Microarray Analysis of Sexual Dimorphism of Adipose Tissues in High-Fat-Diet-Induced Obese Mice. Int J Obes (2010) 34:989–1000. doi: 10.1038/ijo.2010.12 PMC3667412 20157318

[82] Wahrenberg H Lönnqvist F Arner P. Mechanisms Underlying Regional Differences in Lipolysis in Human Adipose Tissue. J Clin Invest (1989) 84:458–67. doi: 10.1172/JCI114187 PMC548904 2503539

[83] Soeters MR Sauerwein HP Groener JE Aerts JM Ackermans MT Glatz JFC. Gender-Related Differences in the Metabolic Response to Fasting. J Clin Endocrinol Metab (2007) 92:3646–52. doi: 10.1210/jc.2007-0552 17566089

[84] Horton TJ Pagliassotti MJ Hobbs K Hill JO. Fuel Metabolism in Men and Women During and After Long-Duration Exercise. J Appl Physiol (1998) 85:1823–32. doi: 10.1152/jappl.1998.85.5.1823 9804587

[85] Bloor ID Symonds ME. Sexual Dimorphism in White and Brown Adipose Tissue With Obesity and Inflammation. Horm Behav (2014) 66:95–103. doi: 10.1016/j.yhbeh.2014.02.007 24589990

[86] Hong J Stubbins RE Smith RR Harvey AE Núñez NP. Differential Susceptibility to Obesity Between Male, Female and Ovariectomized Female Mice. Nutr J (2009) 8:11. doi: 10.1186/1475-2891-8-11 19220919 PMC2650703

[87] Rogers NH Perfield JW Strissel KJ Obin MS Greenberg AS. Reduced Energy Expenditure and Increased Inflammation Are Early Events in the Development of Ovariectomy-Induced Obesity. Endocrinology (2009) 150:2161–8. doi: 10.1210/en.2008-1405 PMC2671894 19179442

[88] Pedersen SB Kristensen K Hermann PA Katzenellenbogen JA Richelsen B. Estrogen Controls Lipolysis by Up-Regulating Alpha2a-Adrenergic Receptors Directly in Human Adipose Tissue Through the Estrogen Receptor Alpha. Implications for the Female Fat Distribution. J Clin Endocrinol Metab (2004) 89:1869–78. doi: 10.1210/jc.2003-031327 15070958

[89] Katzer K Hill JL McIver KB Foster MT. Lipedema and the Potential Role of Estrogen in Excessive Adipose Tissue Accumulation. Int J Mol Sci (2021) 22:11720. doi: 10.3390/ijms222111720 34769153 PMC8583809

[90] Okura T Koda M Ando F Niino N Ohta S Shimokata H. Association of Polymorphisms in the Estrogen Receptor Alpha Gene With Body Fat Distribution. Int J Obes Relat Metab Disord J Int Assoc Study Obes (2003) 27:1020–7. doi: 10.1038/sj.ijo.0802378 12917706

[91] Fletcher LA Kim K Leitner BP Cassimatis TM O’Mara AE Johnson JW. Sexual Dimorphisms in Adult Human Brown Adipose Tissue. Obesity (2020) 28:241–6. doi: 10.1002/oby.22698 PMC6986330 31970907

[92] Herz CT Kulterer OC Prager M Marculescu R Langer FB Prager G. Sex Differences in Brown Adipose Tissue Activity and Cold-Induced Thermogenesis. Mol Cell Endocrinol (2021) 534:111365. doi: 10.1016/j.mce.2021.111365 34126190

[93] Fuller-Jackson J-P Dordevic AL Clarke IJ Henry BA. Effect of Sex and Sex Steroids on Brown Adipose Tissue Heat Production in Humans. Eur J Endocrinol (2020) 183:343–55. doi: 10.1530/EJE-20-0184 32508310

[94] Valle A García-Palmer F Oliver J Roca P. Sex Differences in Brown Adipose Tissue Thermogenic Features During Caloric Restriction. Cell Physiol Biochem (2007) 19:195–204. doi: 10.1159/000099207 17310113

[95] MacCannell ADV Futers TS Whitehead A Moran A Witte KK Roberts LD. Sexual Dimorphism in Adipose Tissue Mitochondrial Function and Metabolic Flexibility in Obesity. Int J Obes 2005 (2021) 45:1773–81. doi: 10.1038/s41366-021-00843-0 PMC8310795 34002038

[96] Cui J Shen Y Li R. Estrogen Synthesis and Signaling Pathways During Ageing: From Periphery to Brain. Trends Mol Med (2013) 19:197–209. doi: 10.1016/j.molmed.2012.12.007 23348042 PMC3595330

[97] Espeland MA Stefanick ML Kritz-Silverstein D Fineberg SE Waclawiw MA James MK. Effect of Postmenopausal Hormone Therapy on Body Weight and Waist and Hip Girths*. J Clin Endocrinol Metab (1997) 82:1549–56. doi: 10.1210/jcem.82.5.3925 9141548

[98] Roesch DM. Effects of Selective Estrogen Receptor Agonists on Food Intake and Body Weight Gain in Rats. Physiol Behav (2006) 87:39–44. doi: 10.1016/j.physbeh.2005.08.035 16181647

[99] Merchenthaler I Lane MV Numan S Dellovade TL. Distribution of Estrogen Receptor α and β in the Mouse Central Nervous System: *In Vivo* Autoradiographic and Immunocytochemical Analyses. J Comp Neurol (2004) 473:270–91. doi: 10.1002/cne.20128 15101093

[100] Heine PA Taylor JA Iwamoto GA Lubahn DB Cooke PS. Increased Adipose Tissue in Male and Female Estrogen Receptor-α Knockout Mice. Proc Natl Acad Sci (2000) 97:12729–34. doi: 10.1073/pnas.97.23.12729 PMC18832 11070086

[101] Yu K He Y Hyseni I Pei Z Yang Y Xu P. 17β-Estradiol Promotes Acute Refeeding in Hungry Mice *via* Membrane-Initiated Erα Signaling. Mol Metab (2020) 42:101053. doi: 10.1016/j.molmet.2020.101053 32712433 PMC7484552

[102] Khbouz B de Bournonville C Court L Taziaux M Corona R Arnal J-F. Role for the Membrane Estrogen Receptor Alpha in the Sexual Differentiation of the Brain. Eur J Neurosci (2020) 52:2627–45. doi: 10.1111/ejn.14646 31833601

[103] Santos RS Frank AP Fátima LA Palmer BF Öz OK Clegg DJ. Activation of Estrogen Receptor Alpha Induces Beiging of Adipocytes. Mol Metab (2018) 18:51–9. doi: 10.1016/j.molmet.2018.09.002 PMC6309577 30270132

[104] Zhou Z Moore TM Drew BG Ribas V Wanagat J Civelek M. Estrogen Receptor α Controls Metabolism in White and Brown Adipocytes by Regulating Polg1 and Mitochondrial Remodeling. Sci Transl Med (2020) 12:eaax8096. doi: 10.1126/scitranslmed.aax8096 32759275 PMC8212422

[105] Ohlsson C Hellberg N Parini P Vidal O Bohlooly M Rudling M. Obesity and Disturbed Lipoprotein Profile in Estrogen Receptor-α-Deficient Male Mice. Biochem Biophys Res Commun (2000) 278:640–5. doi: 10.1006/bbrc.2000.3827 11095962

[106] Santollo J Wiley MD Eckel LA. Acute Activation of Erα Decreases Food Intake, Meal Size, and Body Weight in Ovariectomized Rats. Am J Physiol-Regul Integr Comp Physiol (2007) 293:R2194–201. doi: 10.1152/ajpregu.00385.2007 17942491

[107] González-Granillo M Savva C Li X Ghosh Laskar M Angelin B Gustafsson J-Å. Selective Estrogen Receptor (ER)β Activation Provokes a Redistribution of Fat Mass and Modifies Hepatic Triglyceride Composition in Obese Male Mice. Mol Cell Endocrinol (2020) 502:110672. doi: 10.1016/j.mce.2019.110672 31811898

[108] Ponnusamy S Tran QT Harvey I Smallwood HS Thiyagarajan T Banerjee S. Pharmacologic Activation of Estrogen Receptor α Increases Mitochondrial Function, Energy Expenditure, and Brown Adipose Tissue. FASEB J (2017) 31:266–81. doi: 10.1096/fj.201600787rr PMC5161516 27733447

[109] Zidon TM Padilla J Fritsche KL Welly RJ McCabe LT Stricklin OE. Effects of Erβ and Erα on OVX-Induced Changes in Adiposity and Insulin Resistance. J Endocrinol (2020) 245:165–78. doi: 10.1530/JOE-19-0321 PMC7391131 32053493

[110] Wang A Luo J Moore W Alkhalidy H Wu L Zhang J. GPR30 Regulates Diet-Induced Adiposity in Female Mice and Adipogenesis *In Vitro* . Sci Rep (2016) 6:34302. doi: 10.1038/srep34302 27698362 PMC5048424

[111] Windahl SH Andersson N Chagin AS Mårtensson UEA Carlsten H Olde B. The Role of the G Protein-Coupled Receptor GPR30 in the Effects of Estrogen in Ovariectomized Mice. Am J Physiol-Endocrinol Metab (2009) 296:E490–6. doi: 10.1152/ajpendo.90691.2008 19088255

[112] Davis KE Carstens EJ Irani BG Gent LM Hahner LM Clegg DJ. Sexually Dimorphic Role of G Protein-Coupled Estrogen Receptor (GPER) in Modulating Energy Homeostasis. Horm Behav (2014) 66:196–207. doi: 10.1016/j.yhbeh.2014.02.004 24560890 PMC4051842

[113] Hadjimarkou MM Vasudevan N. GPER1/GPR30 in the Brain: Crosstalk With Classical Estrogen Receptors and Implications for Behavior. J Steroid Biochem Mol Biol (2018) 176:57–64. doi: 10.1016/j.jsbmb.2017.04.012 28465157

[114] Mårtensson UEA Salehi SA Windahl S Gomez MF Swärd K Daszkiewicz-Nilsson J. Deletion of the G Protein-Coupled Receptor 30 Impairs Glucose Tolerance, Reduces Bone Growth, Increases Blood Pressure, and Eliminates Estradiol-Stimulated Insulin Release in Female Mice. Endocrinology (2009) 150:687–98. doi: 10.1210/en.2008-0623 18845638

[115] Sharma G Hu C Brigman JL Zhu G Hathaway HJ Prossnitz ER. GPER Deficiency in Male Mice Results in Insulin Resistance, Dyslipidemia, and a Proinflammatory State. Endocrinology (2013) 154:4136–45. doi: 10.1210/en.2013-1357 PMC3800768 23970785

[116] Sharma G Hu C Staquicini DI Brigman JL Liu M Mauvais-Jarvis F. Preclinical Efficacy of the GPER-Selective Agonist G-1 in Mouse Models of Obesity and Diabetes. Sci Transl Med (2020) 12:eaau5956. doi: 10.1126/scitranslmed.aau5956 31996464 PMC7083206

[117] Qiu J Bosch MA Zhang C Rønnekleiv OK Kelly MJ. Estradiol Protects Neuropeptide Y/Agouti-Related Peptide Neurons Against Insulin Resistance in Females. Neuroendocrinology (2020) 110:105–18. doi: 10.1159/000501560 PMC6920578 31212279

[118] Smith AW Bosch MA Wagner EJ Rønnekleiv OK Kelly MJ. The Membrane Estrogen Receptor Ligand STX Rapidly Enhances GABAergic Signaling in NPY/AgRP Neurons: Role in Mediating the Anorexigenic Effects of 17β-Estradiol. Am J Physiol-Endocrinol Metab (2013) 305:E632–40. doi: 10.1152/ajpendo.00281.2013 PMC3761166 23820624

[119] Smith A Rønnekleiv O Kelly M. Gq-mER Signaling has Opposite Effects on Hypothalamic Orexigenic and Anorexigenic Neurons. Steroids (2014) 0:31–5. doi: 10.1016/j.steroids.2013.11.007 PMC3951396 24269736

[120] Hu P Liu J Yasrebi A Gotthardt JD Bello NT Pang ZP. Gq Protein-Coupled Membrane-Initiated Estrogen Signaling Rapidly Excites Corticotropin-Releasing Hormone Neurons in the Hypothalamic Paraventricular Nucleus in Female Mice. Endocrinology (2016) 157:3604–20. doi: 10.1210/en.2016-1191 PMC5007888 27387482

[121] Roepke TA Bosch MA Rick EA Lee B Wagner EJ Seidlova-Wuttke D. Contribution of a Membrane Estrogen Receptor to the Estrogenic Regulation of Body Temperature and Energy Homeostasis. Endocrinology (2010) 151:4926–37. doi: 10.1210/en.2010-0573 PMC2946146 20685867

[122] Qiu J Bosch MA Tobias SC Krust A Graham SM Murphy SJ. A G-Protein-Coupled Estrogen Receptor Is Involved in Hypothalamic Control of Energy Homeostasis. J Neurosci (2006) 26:5649–55. doi: 10.1523/JNEUROSCI.0327-06.2006 PMC2678732 16723521

[123] Qiu J Rønnekleiv OK Kelly MJ. Modulation of Hypothalamic Neuronal Activity Through a Novel G-Protein-Coupled Estrogen Membrane Receptor. Steroids (2008) 73:985–91. doi: 10.1016/j.steroids.2007.11.008 PMC5466077 18342349

[124] Zhu L Xu P Cao X Yang Y Hinton AO Xia Y. The Erα-PI3K Cascade in Proopiomelanocortin Progenitor Neurons Regulates Feeding and Glucose Balance in Female Mice. Endocrinology (2015) 156:4474–91. doi: 10.1210/en.2015-1660 PMC4655219 26375425

[125] Olofsson LE Pierce AA Xu AW. Functional Requirement of AgRP and NPY Neurons in Ovarian Cycle-Dependent Regulation of Food Intake. Proc Natl Acad Sci (2009) 106:15932–7. doi: 10.1073/pnas.0904747106 PMC2747221 19805233

[126] Gao Q Mezei G Nie Y Rao Y Choi CS Bechmann I. Anorectic Estrogen Mimics Leptin’s Effect on the Rewiring of Melanocortin Cells and Stat3 Signaling in Obese Animals. Nat Med (2007) 13:89–94. doi: 10.1038/nm1525 17195839

[127] Padilla SL Reef D Zeltser LM. Defining POMC Neurons Using Transgenic Reagents: Impact of Transient Pomc Expression in Diverse Immature Neuronal Populations. Endocrinology (2012) 153:1219–31. doi: 10.1210/en.2011-1665 PMC3281533 22166984

[128] Liu T Xu Y Yi C-X Tong Q Cai D. The Hypothalamus for Whole-Body Physiology: From Metabolism to Aging. Protein Cell (2021) 13:394–421. doi: 10.1007/s13238-021-00834-x 33826123 PMC9095790

[129] Asarian L Geary N. Estradiol Enhances Cholecystokinin-Dependent Lipid-Induced Satiation and Activates Estrogen Receptor-α-Expressing Cells in the Nucleus Tractus Solitarius of Ovariectomized Rats. Endocrinology (2007) 148:5656–66. doi: 10.1210/en.2007-0341 17823256

[130] Estacio MA C Tsukamura H Yamada S Tsukahara S Hirunagi K Maeda K. Vagus Nerve Mediates the Increase in Estrogen Receptors in the Hypothalamic Paraventricular Nucleus and Nucleus of the Solitary Tract During Fasting in Ovariectomized Rats. Neurosci Lett (1996) 208:25–8. doi: 10.1016/0304-3940(96)12534-9 8731166

[131] Spary EJ Maqbool A Batten TFC. Changes in Oestrogen Receptor α Expression in the Nucleus of the Solitary Tract of the Rat Over the Oestrous Cycle and Following Ovariectomy. J Neuroendocrinol (2010) 22:492–502. doi: 10.1111/j.1365-2826.2010.01977.x 20236229

[132] Cao X Xu P Oyola MG Xia Y Yan X Saito K. Estrogens Stimulate Serotonin Neurons to Inhibit Binge-Like Eating in Mice. J Clin Invest (2014) 124:4351–62. doi: 10.1172/JCI74726 PMC4191033 25157819

[133] Dalmasso C Amigone JL Vivas L. Serotonergic System Involvement in the Inhibitory Action of Estrogen on Induced Sodium Appetite in Female Rats. Physiol Behav (2011) 104:398–407. doi: 10.1016/j.physbeh.2011.04.029 21554894

[134] Santollo J Torregrossa A-M Eckel LA. Estradiol Acts in the Medial Preoptic Area, Arcuate Nucleus, and Dorsal Raphe Nucleus to Reduce Food Intake in Ovariectomized Rats. Horm Behav (2011) 60:86–93. doi: 10.1016/j.yhbeh.2011.03.009 21439964 PMC3112293

[135] Wang W Wang Z Bai W Zhang H Ma X Yang M. Effect of Low Estrogen on Neurons in the Preoptic Area of Hypothalamus of Ovariectomized Rats. Acta Histochem (2014) 116:1259–69. doi: 10.1016/j.acthis.2014.07.010 25147136

[136] Zhang Z Reis FMCV He Y Park JW DiVittorio JR Sivakumar N. Estrogen-Sensitive Medial Preoptic Area Neurons Coordinate Torpor in Mice. Nat Commun (2020) 11:6378. doi: 10.1038/s41467-020-20050-1 33311503 PMC7732979

[137] Zhang Z Park JW Ahn IS Diamante G Sivakumar N Arneson D. Estrogen Receptor Alpha in the Brain Mediates Tamoxifen-Induced Changes in Physiology in Mice. eLife (2021) 10:e63333. doi: 10.7554/eLife.63333 33647234 PMC7924955

[138] Mori H Matsuda K Pfaff DW Kawata M. A Recently Identified Hypothalamic Nucleus Expressing Estrogen Receptor α. Proc Natl Acad Sci USA (2008) 105:13632–7. doi: 10.1073/pnas.0806503105 PMC2533241 18757761

[139] Balabanov IE Matsuda KI Mori H Yamada S Kitagawa K Yamamoto Y. Neuronal Activity in the Sagittalis Nucleus of the Hypothalamus After Ovarian Steroid Hormone Manipulation and Sexual Behavior in Female Rat. Neurosci Lett (2018) 671:25–8. doi: 10.1016/j.neulet.2018.02.008 29421537

[140] Matsuda KI Uchiyama K Mori H Maejima S Yamaguchi S Tanaka M. Sexual Behavior-Associated C-Fos Induction in the Sagittalis Nucleus of the Hypothalamus in Male Rat. Neurosci Lett (2017) 661:104–7. doi: 10.1016/j.neulet.2017.09.053 28965932

[141] Friedman J. The Long Road to Leptin. J Clin Invest (2016) 126:4727–34. doi: 10.1172/JCI91578 PMC5127673 27906690

[142] Gao Q Horvath TL. Cross-Talk Between Estrogen and Leptin Signaling in the Hypothalamus. Am J Physiol-Endocrinol Metab (2008) 294:E817–26. doi: 10.1152/ajpendo.00733.2007 18334610

[143] Halaas JL Gajiwala KS Maffei M Cohen SL Chait BT Rabinowitz D. Weight-Reducing Effects of the Plasma Protein Encoded by the Obese Gene. Science (1995) 269:543–6. doi: 10.1126/science.7624777 7624777

[144] Hirosawa M Minata M Harada KH Hitomi T Krust A Koizumi A. Ablation of Estrogen Receptor Alpha (Erα) Prevents Upregulation of POMC by Leptin and Insulin. Biochem Biophys Res Commun (2008) 371:320–3. doi: 10.1016/j.bbrc.2008.04.073 18439911

[145] Meli R Pacilio M Raso GM Esposito E Coppola A Nasti A. Estrogen and Raloxifene Modulate Leptin and Its Receptor in Hypothalamus and Adipose Tissue From Ovariectomized Rats. Endocrinology (2004) 145:3115–21. doi: 10.1210/en.2004-0129 15059958

[146] Tanaka M Nakaya S Kumai T Watanabe M Tateishi T Shimizu H. Effects of Estrogen on Serum Leptin Levels and Leptin mRNA Expression in Adipose Tissue in Rats. Horm Res (2001) 56:98–104. doi: 10.1159/000048099 11847470

[147] Del Bianco-Borges B Cabral FJ Franci CR. Co-Expression of Leptin and Oestrogen Receptors in the Preoptic-Hypothalamic Area. J Neuroendocrinol (2010) 22:996–1003. doi: 10.1111/j.1365-2826.2010.02046.x 20584107

[148] Huang K-P Ronveaux CC de Lartigue G Geary N Asarian L Raybould HE. Deletion of Leptin Receptors in Vagal Afferent Neurons Disrupts Estrogen Signaling, Body Weight, Food Intake and Hormonal Controls of Feeding in Female Mice. Am J Physiol-Endocrinol Metab (2019) 316:E568–77. doi: 10.1152/ajpendo.00296.2018 PMC6482667 30753113

[149] Caron A Lee S Elmquist JK Gautron L. Leptin and Brain–Adipose Crosstalks. Nat Rev Neurosci (2018) 19:153–65. doi: 10.1038/nrn.2018.7 PMC5962962 29449715

[150] Fischer AW Hoefig CS Abreu-Vieira G de Jong JMA Petrovic N Mittag J. Leptin Raises Defended Body Temperature Without Activating Thermogenesis. Cell Rep (2016) 14:1621–31. doi: 10.1016/j.celrep.2016.01.041 26876182

[151] Ukropec J Anunciado RVP Ravussin Y Kozak LP. Leptin Is Required for Uncoupling Protein-1-Independent Thermogenesis During Cold Stress. Endocrinology (2006) 147:2468–80. doi: 10.1210/en.2005-1216 16469807

[152] Scarpace PJ Matheny M. Leptin Induction of UCP1 Gene Expression is Dependent on Sympathetic Innervation. Am J Physiol (1998) 275:E259–264. doi: 10.1152/ajpendo.1998.275.2.E259 9688627

[153] Hoffmann A Ebert T Hankir MK Flehmig G Klöting N Jessnitzer B. Leptin Improves Parameters of Brown Adipose Tissue Thermogenesis in Lipodystrophic Mice. Nutrients (2021) 13:2499. doi: 10.3390/nu13082499 34444659 PMC8399124

[154] Shin J-H Hur J-Y Seo HS Jeong Y-A Lee JK Oh M-J. The Ratio of Estrogen Receptor α to Estrogen Receptor β in Adipose Tissue is Associated With Leptin Production and Obesity. Steroids (2007) 72:592–9. doi: 10.1016/j.steroids.2007.03.013 17509633

[155] Yokota-Nakagi N Takahashi H Kawakami M Takamata A Uchida Y Morimoto K. Estradiol Replacement Improves High-Fat Diet-Induced Obesity by Suppressing the Action of Ghrelin in Ovariectomized Rats. Nutrients (2020) 12:907. doi: 10.3390/nu12040907 PMC7230822 32224927

[156] Tanida M Yamamoto N Shibamoto T Rahmouni K. Involvement of Hypothalamic AMP-Activated Protein Kinase in Leptin-Induced Sympathetic Nerve Activation. PloS One (2013) 8:e56660. doi: 10.1371/journal.pone.0056660 23418591 PMC3572050

[157] Lee JH Lin L Xu P Saito K Wei Q Meadows AG. Neuronal Deletion of Ghrelin Receptor Almost Completely Prevents Diet-Induced Obesity. Diabetes (2016) 65:2169–78. doi: 10.2337/db15-1587 PMC4955988 27207529

[158] Matsubara M Sakata I Wada R Yamazaki M Inoue K Sakai T. Estrogen Modulates Ghrelin Expression in the Female Rat Stomach. Peptides (2004) 25:289–97. doi: 10.1016/j.peptides.2003.12.020 15063011

[159] Sakurazawa N Mano-Otagiri A Nemoto T Shibasaki T. Effects of Intracerebroventricular Ghrelin on Food Intake and Fos Expression in the Arcuate Nucleus of the Hypothalamus in Female Rats Vary With Estrous Cycle Phase. Neurosci Lett (2013) 541:204–8. doi: 10.1016/j.neulet.2013.02.006 23435434

[160] Lin L Saha PK Ma X Henshaw IO Shao L Chang BHJ. Ablation of Ghrelin Receptor Reduces Adiposity and Improves Insulin Sensitivity During Aging by Regulating Fat Metabolism in White and Brown Adipose Tissues. Aging Cell (2011) 10:996–1010. doi: 10.1111/j.1474-9726.2011.00740.x 21895961 PMC3215833

[161] Shimobayashi M Albert V Woelnerhanssen B Frei IC Weissenberger D Meyer-Gerspach AC. Insulin Resistance Causes Inflammation in Adipose Tissue. J Clin Invest (2018) 128:1538–50. doi: 10.1172/JCI96139 PMC5873875 29528335

[162] Haczeyni F Bell-Anderson KS Farrell GC. Causes and Mechanisms of Adipocyte Enlargement and Adipose Expansion. Obes Rev Off J Int Assoc Study Obes (2018) 19:406–20. doi: 10.1111/obr.12646 29243339

[163] Wen J Cai X Zhang J Jiang J Li W Liu G. Relation of Adipose Tissue Insulin Resistance to Prediabetes. Endocrine (2020) 68:93–102. doi: 10.1007/s12020-020-02186-8 31925734

[164] Cao W Shi M Wu L Li J Yang Z Liu Y. Adipocytes Initiate an Adipose-Cerebral-Peripheral Sympathetic Reflex to Induce Insulin Resistance During High-Fat Feeding. Clin Sci (2019) 133:1883–99. doi: 10.1042/CS20190412 31477624

[165] Hernández EÁ Kahl S Seelig A Begovatz P Irmler M Kupriyanova Y. Acute Dietary Fat Intake Initiates Alterations in Energy Metabolism and Insulin Resistance. J Clin Invest (2017) 127:695–708. doi: 10.1172/JCI89444 28112681 PMC5272194

[166] Raji A Seely EW Arky RA Simonson DC. Body Fat Distribution and Insulin Resistance in Healthy Asian Indians and Caucasians. J Clin Endocrinol Metab (2001) 86:5366–71. doi: 10.1210/jcem.86.11.7992 11701707

[167] Belsham DD Dalvi PS. Insulin Signalling in Hypothalamic Neurones. J Neuroendocrinol (2021) 33:e12919. doi: 10.1111/jne.12919 33227171

[168] Choi Y-H Fujikawa T Lee J Reuter A Kim KW. Revisiting the Ventral Medial Nucleus of the Hypothalamus: The Roles of SF-1 Neurons in Energy Homeostasis. Front Neurosci (2013) 7:71. doi: 10.3389/fnins.2013.00071 23675313 PMC3646253

[169] Choi SB Jang JS Park S. Estrogen and Exercise May Enhance β-Cell Function and Mass *via* Insulin Receptor Substrate 2 Induction in Ovariectomized Diabetic Rats. Endocrinology (2005) 146:4786–94. doi: 10.1210/en.2004-1653 16037383

[170] Pratchayasakul W Chattipakorn N Chattipakorn SC. Estrogen Restores Brain Insulin Sensitivity in Ovariectomized Non-Obese Rats, But Not in Ovariectomized Obese Rats. Metab Clin Exp (2014) 63:851–9. doi: 10.1016/j.metabol.2014.03.009 24742706

[171] Song D Arikawa E Galipeau DM Yeh JN Battell ML Yuen VG. Chronic Estrogen Treatment Modifies Insulin-Induced Insulin Resistance and Hypertension in Ovariectomized Rats*. Am J Hypertens (2005) 18:1189–94. doi: 10.1016/j.amjhyper.2005.04.003 16182108

[172] Yuan T Li J Zhao W-G Fu Y Liu S-N Liu Q. Effects of Estrogen on Insulin Sensitivity and Adipokines in Mice. Zhongguo Yi Xue Ke Xue Yuan Xue Bao (2015) 37:269–73. doi: 10.3881/j.issn.1000-503X.2015.03.004 26149135

[173] Felig P. Insulin is the Mediator of Feeding-Related Thermogenesis: Insulin Resistance and/or Deficiency Results in a Thermogenic Defect Which Contributes to the Pathogenesis of Obesity. Clin Physiol Oxf Engl (1984) 4:267–73. doi: 10.1111/j.1475-097x.1984.tb00802.x 6380904

[174] Peirce V Vidal-Puig A. Regulation of Glucose Homoeostasis by Brown Adipose Tissue. Lancet Diabetes Endocrinol (2013) 1:353–60. doi: 10.1016/S2213-8587(13)70055-X 24622420

[175] Orava J Nuutila P Lidell ME Oikonen V Noponen T Viljanen T. Different Metabolic Responses of Human Brown Adipose Tissue to Activation by Cold and Insulin. Cell Metab (2011) 14:272–9. doi: 10.1016/j.cmet.2011.06.012 21803297

[176] Chondronikola M Volpi E Børsheim E Porter C Annamalai P Enerbäck S. Brown Adipose Tissue Improves Whole-Body Glucose Homeostasis and Insulin Sensitivity in Humans. Diabetes (2014) 63:4089–99. doi: 10.2337/db14-0746 PMC4238005 25056438

[177] Benedict C Brede S Schiöth HB Lehnert H Schultes B Born J. Intranasal Insulin Enhances Postprandial Thermogenesis and Lowers Postprandial Serum Insulin Levels in Healthy Men. Diabetes (2010) 60:114–8. doi: 10.2337/db10-0329 PMC3012162 20876713

[178] Maliszewska K Kretowski A. Brown Adipose Tissue and Its Role in Insulin and Glucose Homeostasis. Int J Mol Sci (2021) 22:1530. doi: 10.3390/ijms22041530 33546400 PMC7913527

[179] Heine M Fischer AW Schlein C Jung C Straub LG Gottschling K. Lipolysis Triggers a Systemic Insulin Response Essential for Efficient Energy Replenishment of Activated Brown Adipose Tissue in Mice. Cell Metab (2018) 28:644–55.e4. doi: 10.1016/j.cmet.2018.06.020 30033199

[180] Guerra C Navarro P Valverde AM Arribas M Brüning J Kozak LP. Brown Adipose Tissue–Specific Insulin Receptor Knockout Shows Diabetic Phenotype Without Insulin Resistance. J Clin Invest (2001) 108:1205–13. doi: 10.1172/JCI13103 PMC209529 11602628

[181] Martínez de Morentin PB Martinez-Sanchez N Roa J Ferno J Nogueiras R Tena-Sempere M. Hypothalamic mTOR: The Rookie Energy Sensor. Curr Mol Med (2014) 14:3–21. doi: 10.2174/1566524013666131118103706 24236459

[182] González-García I Martínez de Morentin PB Estévez-Salguero Á Contreras C Romero-Picó A Fernø J. mTOR Signaling in the Arcuate Nucleus of the Hypothalamus Mediates the Anorectic Action of Estradiol. J Endocrinol (2018) 238:177–86. doi: 10.1530/JOE-18-0190 PMC6055430 29914932

[183] Romero-Picó A Novelle MG Al-Massadi O Beiroa D Tojo M Heras V. Kappa-Opioid Receptor Blockade Ameliorates Obesity Caused by Estrogen Withdrawal *via* Promotion of Energy Expenditure Through mTOR Pathway. Int J Mol Sci (2022) 23:3118. doi: 10.3390/ijms23063118 35328539 PMC8953356

[184] Hetherington AW Ranson SW. Hypothalamic Lesions and Adiposity in the Rat. Anat Rec (1940) 78:149–72. doi: 10.1002/ar.1090780203

[185] Perkins MN Rothwell NJ Stock MJ Stone TW. Activation of Brown Adipose Tissue Thermogenesis by the Ventromedial Hypothalamus. Nature (1981) 289:401–2. doi: 10.1038/289401a0 7464907

[186] Lindberg D Chen P Li C. Conditional Viral Tracing Reveals That Steroidogenic Factor 1-Positive Neurons of the Dorsomedial Subdivision of the Ventromedial Hypothalamus Project to Autonomic Centers of the Hypothalamus and Hindbrain. J Comp Neurol (2013) 521:3167–90. doi: 10.1002/cne.23338 23696474

[187] Rau AR Hentges ST. Energy State Alters Regulation of Proopiomelanocortin Neurons by Glutamatergic Ventromedial Hypothalamus Neurons: Pre- and Postsynaptic Mechanisms. J Neurophysiol (2021) 125:720–30. doi: 10.1152/jn.00359.2020 PMC7988752 33441043

[188] He Y Xu P Wang C Xia Y Yu M Yang Y. Estrogen Receptor-α Expressing Neurons in the Ventrolateral VMH Regulate Glucose Balance. Nat Commun (2020) 11:2165. doi: 10.1038/s41467-020-15982-7 32358493 PMC7195451

[189] López M Nogueiras R Tena-Sempere M Diéguez C. Hypothalamic AMPK: A Canonical Regulator of Whole-Body Energy Balance. Nat Rev Endocrinol (2016) 12:421–32. doi: 10.1038/nrendo.2016.67 27199291

[190] Martins L Seoane-Collazo P Contreras C González-García I Martínez-Sánchez N González F. A Functional Link Between AMPK and Orexin Mediates the Effect of BMP8B on Energy Balance. Cell Rep (2016) 16:2231–42. doi: 10.1016/j.celrep.2016.07.045 PMC4999418 27524625

[191] Beiroa D Imbernon M Gallego R Senra A Herranz D Villarroya F. GLP-1 Agonism Stimulates Brown Adipose Tissue Thermogenesis and Browning Through Hypothalamic AMPK. Diabetes (2014) 63:3346–58. doi: 10.2337/db14-0302 24917578

[192] Martínez-Sánchez N Moreno-Navarrete JM Contreras C Rial-Pensado E Fernø J Nogueiras R. Thyroid Hormones Induce Browning of White Fat. J Endocrinol (2017) 232:351–62. doi: 10.1530/JOE-16-0425 PMC5292977 27913573

[193] Liu H Xu Y Hu F. AMPK in the Ventromedial Nucleus of the Hypothalamus: A Key Regulator for Thermogenesis. Front Endocrinol (2020) 11:578830. doi: 10.3389/fendo.2020.578830 PMC7538541 33071984

[194] Valeria Torres Irizarry. BioRender.com (Toronto, CA). E2-VMH-AMPK-SNS-BAT axis. (2022) Created with BioRender.com.

[195] Ye H Feng B Wang C Saito K Yang Y Ibrahimi L. An Estrogen-Sensitive Hypothalamus-Midbrain Neural Circuit Controls Thermogenesis and Physical Activity. Sci Adv (2022) 8:eabk0185. doi: 10.1126/sciadv.abk0185 35044814 PMC8769556

[196] Heyder N Kleinau G Szczepek M Kwiatkowski D Speck D Soletto L. Transduction and Pathogenic Modifications at the Melanocortin-4 Receptor: A Structural Perspective. Front Endocrinol (2019) 10:515. doi: 10.3389/fendo.2019.00515 PMC6685040 31417496

[197] Bartness TJ Liu Y Shrestha YB Ryu V. Neural Innervation of White Adipose Tissue and the Control of Lipolysis. Front Neuroendocrinol (2014) 35:473–93. doi: 10.1016/j.yfrne.2014.04.001 PMC4175185 24736043

[198] Hirschberg PR Sarkar P Teegala SB Routh VH. Ventromedial Hypothalamus Glucose-Inhibited Neurones: A Role in Glucose and Energy Homeostasis? J Neuroendocrinol (2020) 32:e12773. doi: 10.1111/jne.12773 31329314 PMC7074896

[199] Steinbusch LKM Picard A Bonnet MS Basco D Labouèbe G Thorens B. Sex-Specific Control of Fat Mass and Counterregulation by Hypothalamic Glucokinase. Diabetes (2016) 65:2920–31. doi: 10.2337/db15-1514 27422385

[200] Jordan SD Könner AC Brüning JC. Sensing the Fuels: Glucose and Lipid Signaling in the CNS Controlling Energy Homeostasis. Cell Mol Life Sci (2010) 67:3255–73. doi: 10.1007/s00018-010-0414-7 PMC2933848 20549539

[201] Santiago AM Clegg DJ Routh VH. Estrogens Modulate Ventrolateral Ventromedial Hypothalamic Glucose-Inhibited Neurons. Mol Metab (2016) 5:823–33. doi: 10.1016/j.molmet.2016.08.002 PMC5034617 27688996

[202] WHO. Obesity (2021). World Health Organ. Available at: https://www.who.int/westernpacific/health-topics/obesity (Accessed April 25, 2021).

